# Synthesis of Phenol-Hydrazide-Appended Tetraphenylethenes as Novel On–Off–On Cascade Sensors of Copper and Glutathione

**DOI:** 10.1021/acsomega.4c02043

**Published:** 2024-06-07

**Authors:** Sinan Bayindir, Sebiha Akar

**Affiliations:** † Department of Chemistry, Faculty of Sciences and Arts, Bingol University, 12000 Bingol, Türkiye; ‡ Department of Chemistry, Graduate School of Natural and Applied Sciences, Bingol University, 12000 Bingol, Türkiye

## Abstract

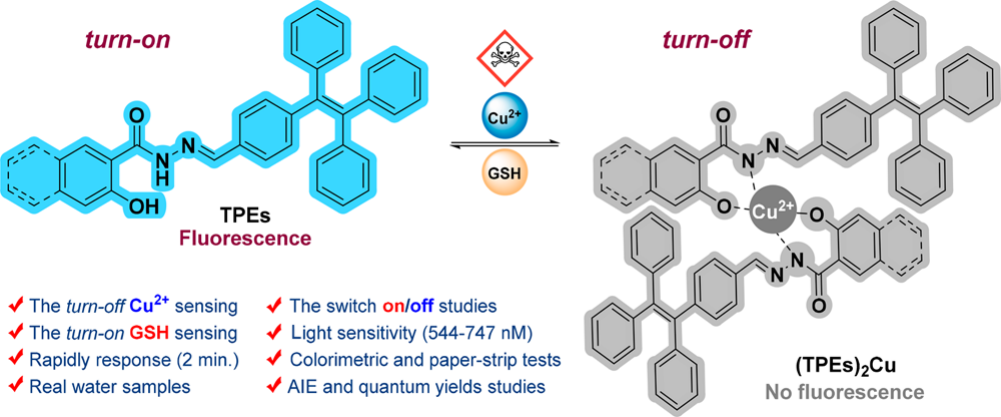

This study reports the synthesis of novel fluorescent probes, phenol-hydrazide-appended tetraphenylethenes (**TPEs I** and **II**), and explores their photochemical properties. The probes exhibit aggregation-induced emission (AIE) in increasing water content, as observed using fluorescence spectroscopy. Further investigation with UV–vis and fluorescence techniques revealed their potential as ion sensors. Both **TPE I** and **TPE II** act as “turn-off” sensors for Cu^2+^ ions, showing decreased fluorescence intensity in their presence. Their limit of detection (LOD) and association constant (*K*_a_) for Cu^2+^ were found to be comparable at 747 nM/597 nM, and 2.46 × 10^5^ M^–1/2^/1.78 × 10^5^ M^–1/2^, respectively. Moreover, the quantum yields of **TPE I** and **TPE II** were also calculated and found to be 0.651 and 0.325, respectively. Interestingly, these probes also function as “turn-on” sensors for glutathione (GSH) in the presence of copper. This means their fluorescence can be reversibly switched off and on by alternating CuCl_2_ and GSH additions. Moreover, the LOD values for GSH with TPE II–Cu^2+^ were calculated to be 544 nM. In addition, the investigation also employed visual analysis to assess the color alterations of TPEs on filter paper and in real water samples. Overall, this research introduces promising new probes with potential applications in copper ion detection and biomolecule glutathione sensing in real water samples.

## 1 Introduction

Nowadays, colorimetric and fluorometric techniques are rapidly becoming popular choices for detecting ions due to their simplicity and sensitivity. These methods offer a viable alternative to more expensive and complex techniques like voltammetry, plasma mass spectrometry, and atomic emission spectrometry. This growing interest highlights the importance of developing simple organic compounds that can selectively target specific metal ions within a mixture of contaminants.^1−5^ Copper, for instance, is a naturally abundant metal crucial for various bodily functions. It works in conjunction with proteins to form enzymes that act as catalysts. The ability to specifically detect copper, along with other metals, in such mixtures becomes especially important.^6,7^ Because research shows that misregulation of copper may contribute to the development of various genetic and metabolic disorders in humans, such as Parkinson’s disease, Wilson’s disease, Alzheimer’s disease, obesity, and diabetes.^8,9^ However, the biochemistry of copper (Cu^2+^) ions in eukaryotic cells is complex, as they serve as essential cofactors for several redox enzymes that interact with dioxygen and its derivatives, such as superoxide.^10,11^ Depending on the exposure level, copper ions, which are crucial for health, can enter the human body through contaminated water sources contaminated by industrial or consumer waste, or through acid rain.^12,13^ Considering the potential health impacts of copper, the Environmental Protection Agency (EPA) identifies Cu^2+^ as a potential trace pollutant and has set a permissible level of copper in drinking water between 1.3 ppm and 20 μM.^14−17^ The average concentration of copper in human blood is around 15.7–23.6 μM.^18,19^ Therefore, monitoring copper levels in drinking water and other environmental sources is essential.^20^ Besides the importance of selective recognition of copper ions, glutathione (GSH), a ubiquitous antioxidant found in living cells, plays a crucial role in maintaining cellular health and protecting against various diseases.^21^ Composed of glutamic acid, cysteine, and glycine, GSH possesses remarkable antioxidative properties, safeguarding the sulfhydryl groups in proteins and enzymes, thereby preserving their redox state.^22,23^ With varying concentrations ranging from 0.5 to 15 mM depending on cell type, GSH levels exhibit significant differences between healthy and cancerous tissues.^24^ Aberrant GSH concentrations have been linked to a spectrum of ailments, including liver damage, Alzheimer’s, Parkinson’s, coronary heart disease, edema, slowed growth, clinical stroke, lung damage, and asthma.^25,26^ The critical role of GSH in maintaining physiological homeostasis underscores the need for sensitive and selective detection methods.^27,28^ Among these approaches, fluorescence-based sensing methods utilizing small molecule probes have emerged as frontrunners due to their simplicity, noninvasive nature, high sensitivity, and excellent reproducibility in biological settings.^29−31^ Moreover, copper and GSH have a strong interaction within water. The GSH, a vital biological molecule, can be affected by copper through oxidation. This interaction is important because GSH acts like a cellular buffer, maintaining a balance between its reduced and oxidized forms. This balance, in turn, regulates the activity of enzymes that rely on thiol groups and are sensitive to changes in oxidation. Interestingly, GSH also forms strong bonds with copper, and this complex can even transport copper within the cell. This explains the strong reversible interaction of GSH with copper in the probes–copper complex.^32^ In addition to the importance of specific detection of Cu^2+^ ions and GSH, aggregation-induced emission (AIE) is a technologically important and remarkable phenomenon that has received much attention since the beginning of the century.^33^ It is particularly useful for the specific detection of ions, which is a crucial area of research. In this context, tetraphenylethene (TPE) is an effective fluorophore that exhibits AIE.^34,35^ Researchers are exploring the use of these molecules for more than just light emission.^36−40^ They can act as energy donors when combined with other fluorescent molecules. Additionally, recent studies have shown promise for using organic materials containing tetraphenylethene (TPE) and hydrazone units as sensors. These materials have been investigated for their ability to detect specific ions with high sensitivity (low limit of detection, LOD) due to the desirable properties of both TPE and hydrazone units. TPE units are favorable because they tend to aggregate effectively, while hydrazone groups are preferred for their strong interactions with ions and the presence of water-soluble groups. In this study, we synthesized two new **TPEs I** and **II**, which incorporate either phenyl or naphthol groups. We chose TPE due to its frequent use in similar research and opted for structurally diverse phenyl and naphthalene-substituted hydrazone units. We then investigated their quantum yields (Φ), AIE properties, and their ability to detect a wide range of ions using colorimetric and spectroscopic techniques.

## 2 Results and Discussions

### 2.1 Chemistry

**Scheme 1:**
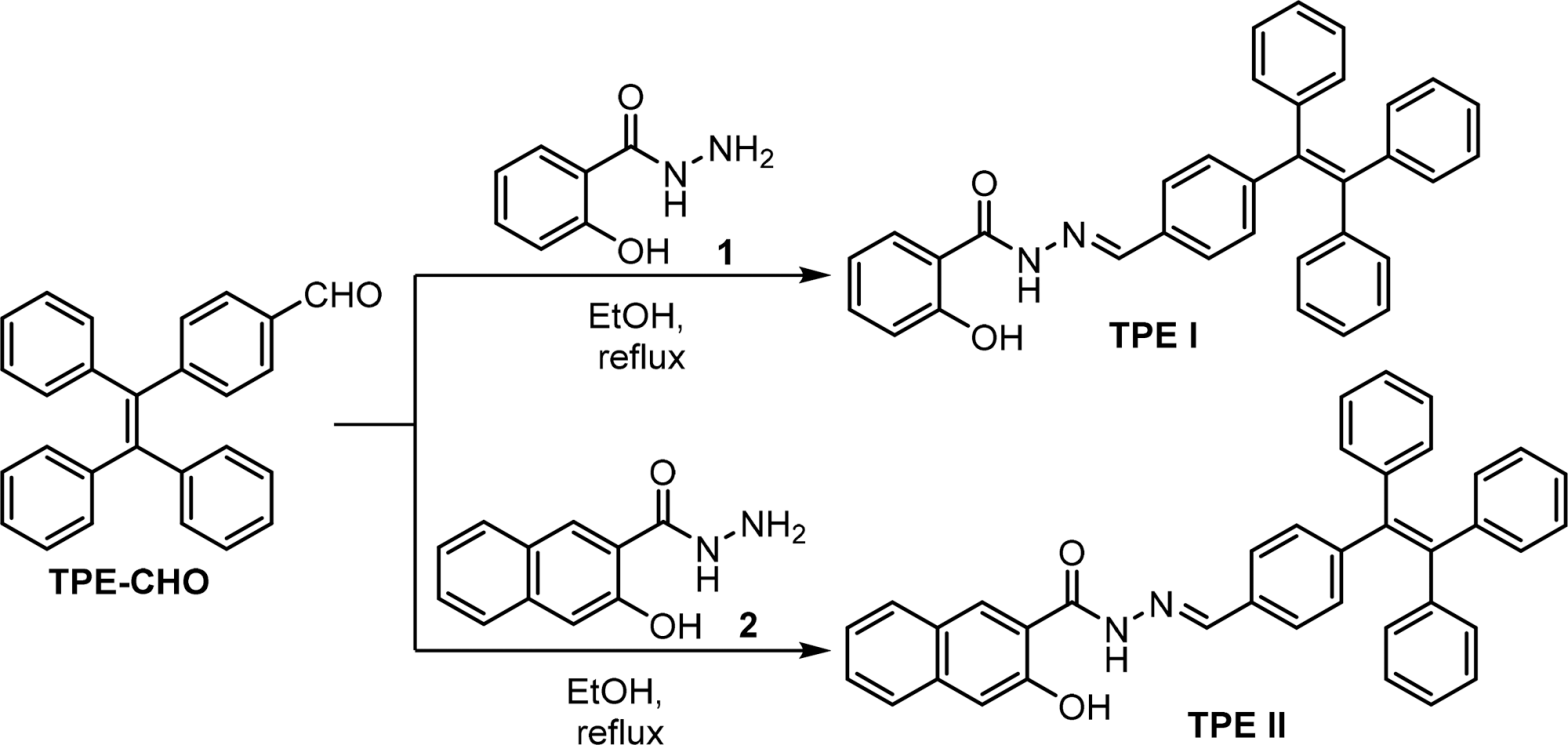
Synthesis Strategy of **TPE I** and **TPE II**

The synthesis of tetraphenylethene (TPE)-based organic compounds and the investigation of their ion sensor properties have been popular research areas in recent years. In this study, we synthesized two novel TPE derivatives, **TPE I** and **TPE II**, using a multistep reaction route (Schemes 1 and S1 of the Supporting Information). First, we synthesized the intermediate compound TPE-CHO according to the literature (Scheme S1).^36^ Then, we reacted TPE-CHO with 2-hydroxybenzohydrazide (**1**, for *TPE I*) or 3-hydroxy-2-naphthohydrazide (**2**, for *TPE II*) in ethanol at reflux temperature to obtain the target derivatives **TPE I** and **TPE II**, respectively.

### 2.2 UV–vis and Fluorescence Response of TPEs to Various Ions

**Figure 1:**
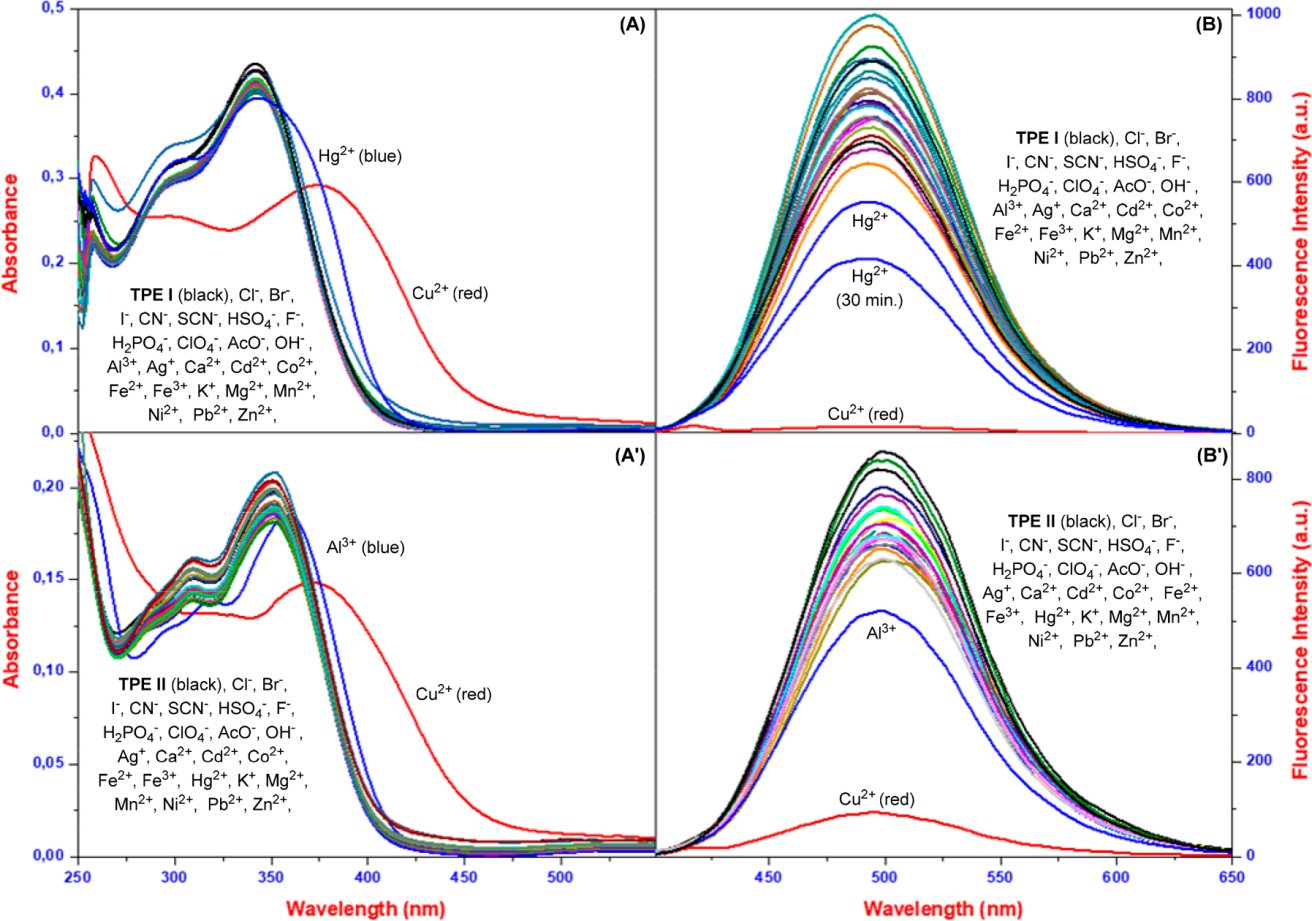
UV–vis and fluorescence of spectra TPE I (**A** and **B**) and TPE II (**A′** and **B′**) in the absence and presence of ions in EtOH/HEPES buffer (v/v:0.1/9.9)

Following the synthesis of **TPE I** and **TPE II**, we investigated the probe’s interaction with various ions by studying the ultraviolet–visible (UV–vis) and fluorescence of the probes in a variety of solvent systems, including MeOH, EtOH, DMSO, THF, and their aqueous counterparts (Figure S5A). In these experiments, the interactions of **TPE I** and TPE II with various metals (Al^3+^, Ag^+^, Ca^2+^, Cd^2+^, Co^2+^, Cu^2+^, Fe^2+^, Fe^3+^, Hg^2+^, K^+^, Mg^2+^, Mn^2+^, Ni^2+^, Pb^2+^, and Zn^2+^ as their chloride salts) and anions ([Bu_4_N]F, [Bu_4_N]Cl, [Bu_4_N]Br, [Bu_4_N]I, [Bu_4_N]AcO, [Bu_4_N]HSO_4_, [Bu_4_N]ClO_4_, [Bu_4_N]CN, [Bu_4_N]SCN, [Bu_4_N]H_2_PO_4_, and [Bu_4_N]OH) were studied in selected solvent systems. The exploration of solvent optimization commenced with anhydrous organic solvents. We found that there was no interaction between the probes and Cu^2+^ ions in MeOH, THF, and DMSO. However, notable spectral changes, manifesting as a color transformation from colorless to yellow in response to Cu^2+^ ions, were observed in EtOH. Similarly, in aqueous solutions, no interactions were noted for MeOH, and THF, but a specific interaction with Cu^2+^ ions emerged in studies involving aqueous solutions of DMSO and EtOH. We also found that the probes interacted specifically with Cu^2+^ ions in pure water and tap water. On the basis of all of these results, we concluded that both probes interact with Cu^2+^ ions in EtOH, EtOH/H_2_O, DMSO/H_2_O, pure water, and real water samples (Figure S5A). Following the initial selection of a solvent system, optimization studies were conducted to determine the optimal water ratio and pH for the probes (Figure S5B). Studies to determine the water ratio were conducted for all ions, and it was observed that the probes interacted exclusively with copper ions. While close interaction values were observed across all water ratios, the most stable and effective results for **TPE I** and **TPE II** were obtained from studies carried out in EtOH/H_2_O (v/v: from 1/1 to 0.1/9.9). The pH studies are crucial for characterizing sensor candidate organic probes. Therefore, following the identification of the optimal water ratio, pH studies were conducted within a range of 2 to 12. Studies on pH levels revealed that the interaction between TPEs and copper ions was weak in both acidic and basic environments. In contrast, interaction was observed at pH 7 and in HEPES buffer environments. Nonetheless, the absorbance spectra of TPEs remained constant across all pH values tested. As a result of optimization studies, despite their important advantages, such as compatibility with real water samples, adaptability to varying water ratios, and reproducible results, the probes’ limited pH range can be considered a drawback. Following solvent and pH studies, the optimization studies were concluded with time studies involving TPEs in the presence of copper ions in EtOH/HEPES. It was found that both probes achieved maximum interaction within approximately 2 min and maintained this interaction over time (Figure S6A). The lack of significant interaction with other ions in long-term interaction studies highlights the probes’ significant advantage in terms of both time and selectivity. A detailed examination of the data revealed that except for studies involving real water samples, the suitable solvent systems for probes were EtOH/HEPES or HEPES solvent systems. The interactions of **TPE I** and **TPE II** with different metals and anions were investigated in EtOH/HEPES (v/v:1/9). UV–vis spectra for all ions were recorded approximately 5 min after the addition of three equivalents of each ion. UV–vis studies in selected solvent systems revealed a decrease in the absorption band of **TPE I**/**TPE II** from 342/350 nm to 378/371 nm in the presence of Cu^2+^, indicating redshifts (Figure 1A/1A′). Additionally, from the absorption studies, no significant change was observed with other anions and cations except copper ions. Beyond UV–vis experiments and characterization studies, understanding the individual interactions of probes with ions and the resulting spectral changes is crucial, as a single ion is unlikely to be present in real-world samples. Therefore, competitive experiments were conducted in EtOH/HEPES to investigate the influence of other ions on the binding of Cu^2+^ ions with the probes **TPE I** and **TPE II**. The results revealed that the absorbance change induced by the mixture of Cu^2+^ with all other ions was similar to that induced by copper alone (Figure S6B). Consequently, none of the tested ions interfered with the interaction of both **TPE I** and **TPE II** with Cu^2+^ in the selected solvent systems and real water samples. In addition to UV–vis spectroscopy, fluorescence spectroscopy was also employed to assess the cation and/or anion-sensing capabilities of **TPE I** and **TPE II**. Fluorescence studies with various ions revealed a specific spectral change in response to Cu^2+^ ions, corroborating the findings of the UV–vis studies. In addition to UV–vis spectroscopy, fluorescence spectroscopy was also employed to assess the cation and/or anion-sensing capabilities of TPEs. Fluorescence studies with various ions revealed a specific spectral change in response to Cu^2+^ ions, corroborating the findings of the UV–vis studies (Figure 1B/1B′). Following preliminary fluorescence studies, detailed fluorescence studies were also carried out to assess the cation and/or anion-sensing capabilities of **TPE I** and **TPE II** in ethanol/HEPES buffer (v/v:0.1/9.9). **TPE I** and **TPE II** displayed emission peaks at 493 and 502 nm upon excitation at 370 nm, respectively. Adding various ions (except Cu^2+^) caused minimal spectral shifts. Importantly, Cu^2+^ interactions led to significant changes: both probes showed decreased fluorescence intensity. This indicates their potential as selective “turn-off” fluorescent sensors for copper ions. Sensitivity toward Cu^2+^ was further assessed with fluorescence titrations in water (excitation at 370 nm). Interestingly, fluorescence studies revealed weak interactions between **TPE I**/**TPE II** and mercury/aluminum ions, respectively. These interactions were measured after a 30 min incubation period. While **TPE II** showed no significant change, **TPE I** displayed a notable decrease in its interaction with mercury ions (Figure 1B). This decrease remained stable over time, suggesting a small interaction event.

**Figure 2:**
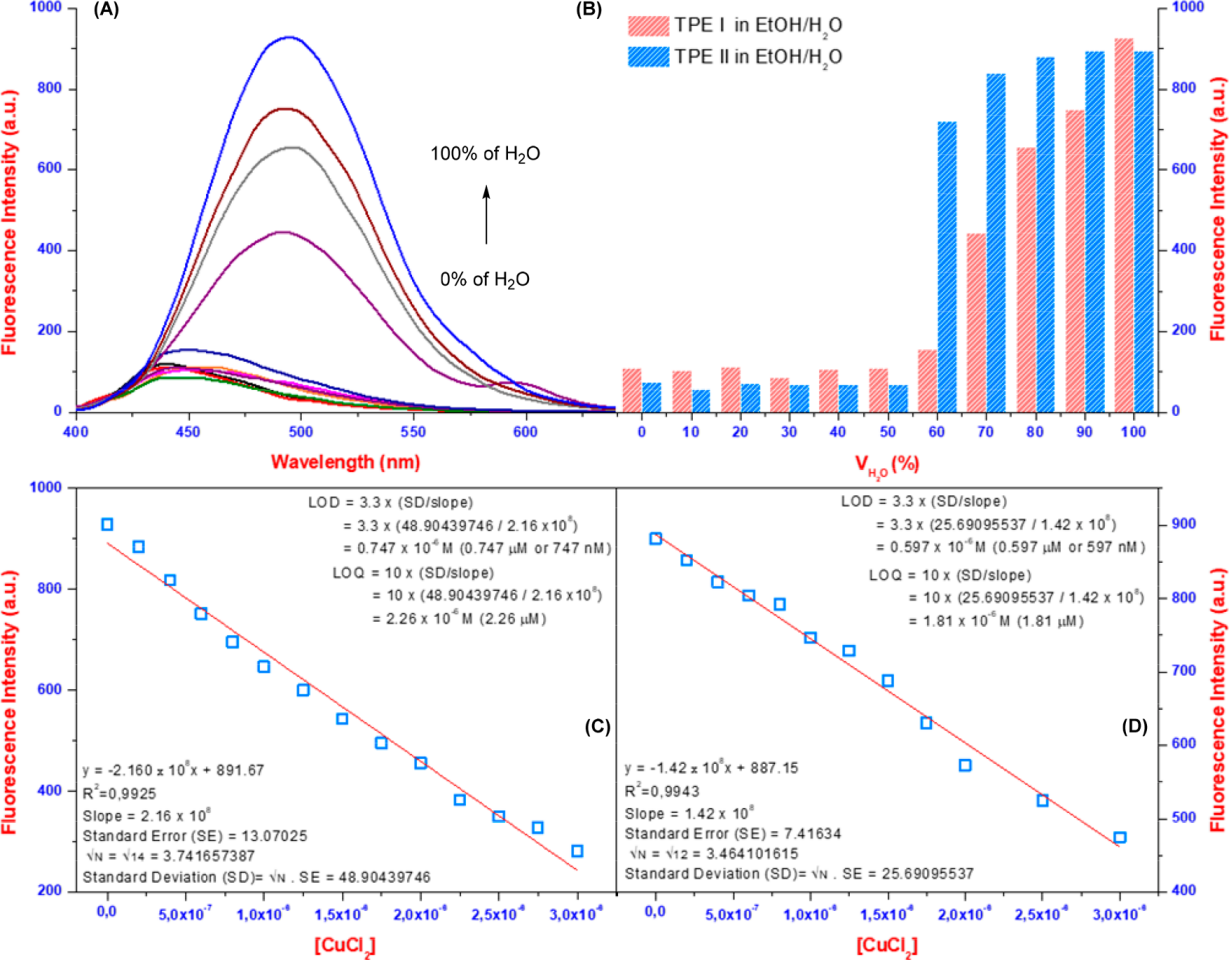
AIE fluorescent response (A) and bar graphic (B) of TPEs in different water ratio mixtures (λ_exc_ = 370 nm), and the change fluorescence intensity of the TPEs TPE I (C) and TPE II (D) with the increasing concentration of CuCl_2_.

Additionally, fluorescence studies following UV–vis analysis revealed a decrease in intensity due to ligand-copper interactions, prompting an AIE investigation (Figures 2A, 2B, and S7). Ethanol was chosen for its safety. However, the TPEs displayed complete fluorescence quenching in pure ethanol due to photoinduced electron transfer (PET) or excited-state intramolecular proton transfer (ESIPT). Interestingly, adding water incrementally (starting with 70%) reversed the quenching, reaching a maximum intensity at 100% water. This significant increase, accompanied by a slight red-shift, confirms the AIE behavior of the TPEs. Suppression of PET due to enhanced aggregation is the proposed mechanism. The presence of nitrogen and hydroxyl groups in the TPEs facilitates electron transfer within the molecule, which aggregation disrupts. Therefore, hydrogen-bonding interactions between water and TPE donors (hydroxyl, imine, or amine) are believed to inhibit PET, resulting in AIE.^40−42^ Similar AIE behavior was observed in TPE-Cu^2+^ complexes with increasing water content, but the intensity increase was much smaller. This suggests hindered aggregation due to the altered structure of the complexes. Moreover, fluorescence titrations of both **TPE I** and **TPE II** were performed in the presence of increasing copper ions (Figure S8). Both TPEs showed gradual fluorescence quenching with increasing CuCl_2_ concentrations, and they reached birth at 4 μM and 7 μM levels, respectively. Following titration studies, the LOD and the limit of quantification (LOQ) values were determined for TPEs using fluorescence titration data and the corresponding eqs 1 and 2). Accordingly, the LOD and LOQ values of **TPE I** and **TPE II** were calculated as 0.747/0.597 μM and 2.26/1.81 μM, respectively (Figure 2C and 2D).
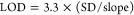
1
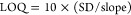
2

**Figure 3:**
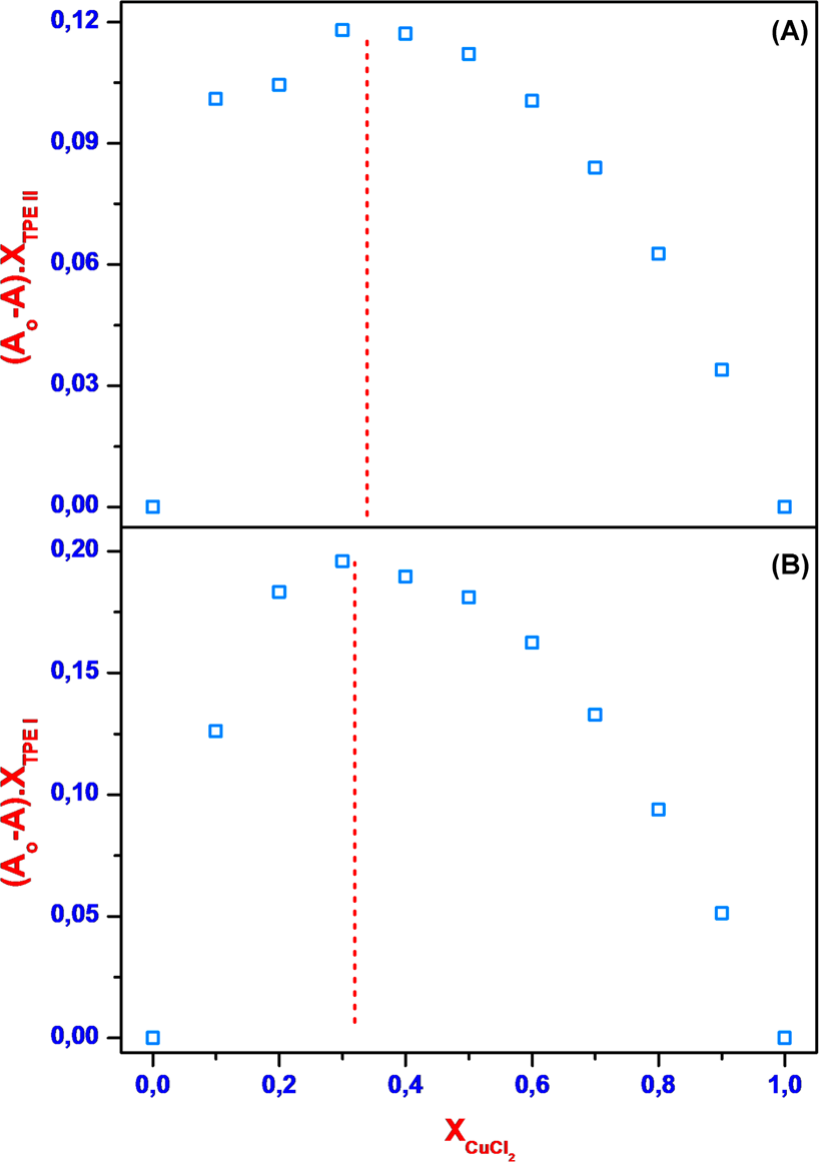
Job’s plot of TPE II (A)/TPE I (B) with CuCl_2_.

Determining the binding constant (*K*_a_) value is crucial for evaluating sensor candidates. *K*_a_ values are calculated by first establishing the stoichiometry of the binding interaction between copper and the organic probes **TPE I** and **TPE II**. Job’s plot experiments, as detailed in the Experimental Section, were conducted to determine the binding stoichiometry between **TPE I** or **TPE II** and Cu^2+^. The absorbance intensity of mixtures containing varying molar ratios of Cu^2+^ and **TPE I** or **TPE II** was measured at room temperature. The results revealed that **TPE I** or **TPE II** form a 2:1 (L_2_M) complex with Cu^2+^ (Figures 3 and S9). The *K*_a_ values of **TPE I** and **TPE II** for Cu^2+^ were determined using the fluorescence titration results and the Benesi–Hildebrand eqs 3. The *K*_a_ values of **TPE I** and **TPE II** with Cu^2+^ were calculated to be 2.46 × 10^5^ M^–1/2^ and 1.78 × 10^5^ M^–1/2^, respectively (Figure S10). These results indicate a relatively strong binding affinity between the probes and copper ions.
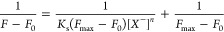
3

**Figure 4:**
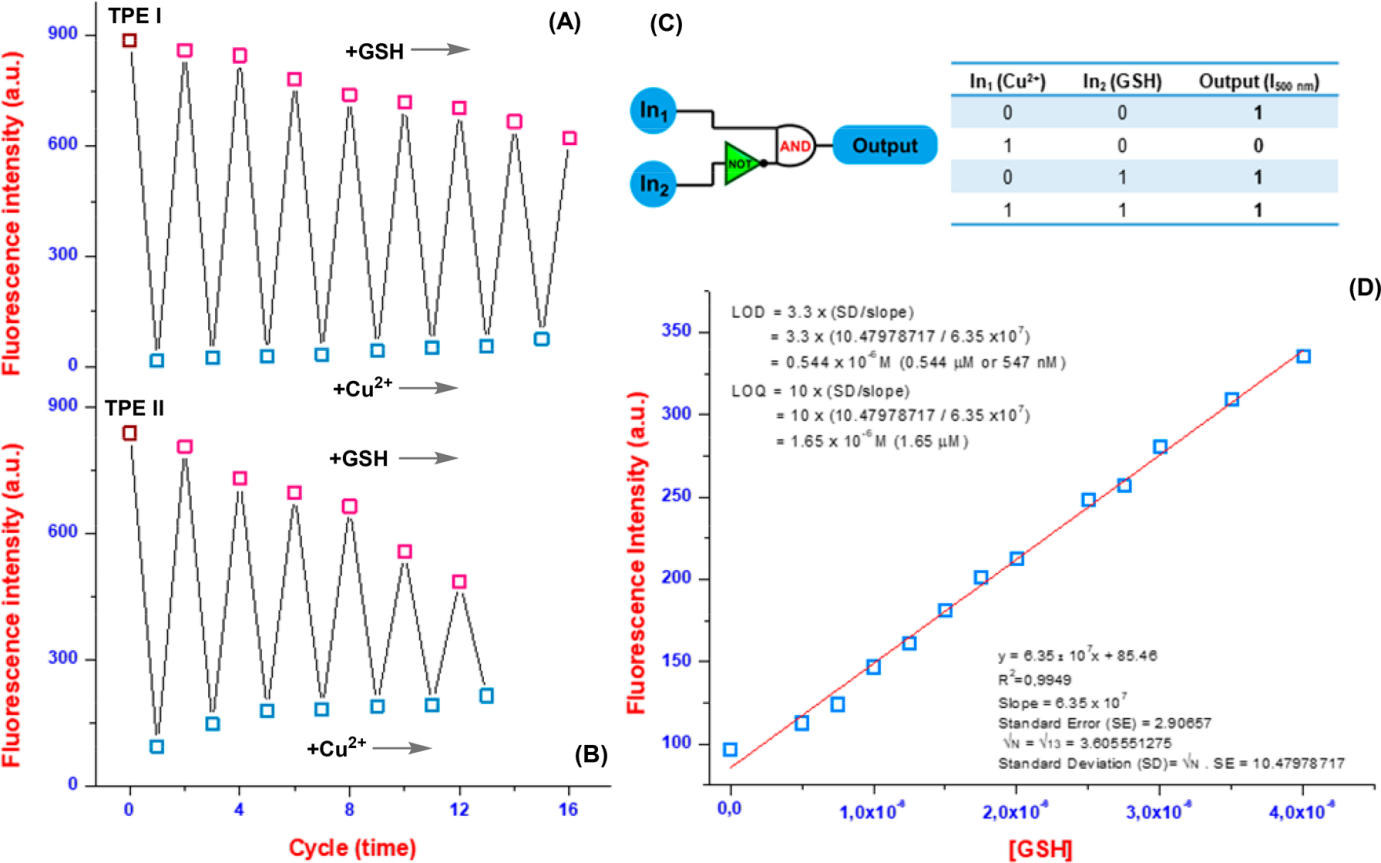
Reversible switching of the fluorescence intensity of TPE I (A) and TPE II (B), and (C) the “IMPLICATION” logic gate. (D) The change in fluorescence intensity of the TPE II–Cu^2+^ with the increasing concentration of GSH.

Organic probes possess another remarkable feature: the switchable or reversible sensing properties of chemosensor candidates. Reversal studies have demonstrated that alternating the addition of CuCl_2_ and GSH to **TPE I** and **TPE II** induces a switchable off/on variation in the emission intensity at around 500 nm. This remarkable property allows **TPE I** and **TPE II** to be readily reused for Cu^2+^ and GSH sensing for up to about 16 and 13 cycles, respectively (Figures 4A, 4B, and S11). Interestingly, the amount of fluorescence decreases as more GSH is added. This suggests that the interaction between TPEs and copper ions leads to a stable complex between GSH and copper ions. Moreover, the optical properties of probes with copper and GSH as inputs to construct a molecular logic function were employed. To establish a logic gate, we assigned the values of “1” and “0” to the fluorescence states of “on” and “off”, respectively. Based on this assignment, when the input data is tailored, the **TPE I** and **TPE II** chemosensors remain in the “on” state in the absence of the inputs Cu^2+^ (In1) and GSH (In2). However, when Cu^2+^ (In1) is added to the probes, a reduction in the emission intensity at around 500 nm is observed, resulting in an output logic of “0”, which corresponds to the “off” state. Conversely, when GSH (In2) is added to TPEs-Cu^2+^, an increase in the emission peak at around 500 nm is noted, leading to an output logic of “1”, which corresponds to the “on” state (Figure 4C). The reversible interaction of GSH ions with the TPEs-Cu^2+^ complex suggested their potential as GSH sensors. Therefore, fluorescence titration studies of the TPEs-Cu^2+^ complex with GSH were performed. As the GSH concentration increased, the fluorescence peak at 498 nm rose proportionally, reaching saturation at approximately 5.0 μM (Figure S12). Using the obtained titration data and relevant formulas, the LOD and LOQ values for GSH were calculated to be 547 nM and 1.65 μM, respectively (Figure 4D). These low LOD and LOQ values demonstrate the complex’s promising potential for sensitive and precise GSH detection.

Additionally, interaction morphology plays a crucial role in sensor studies. On the basis of existing literature, three potential binding sites in TPEs are identified for copper ions: the N atom in the Schiff base, the OH groups of the Ph/Np group, and the NH core of the linker. ^1^H NMR spectroscopy was not suitable for studying the interaction between TPEs and Cu^2+^ ions due to the complex’s paramagnetism. Therefore, FT-IR measurements were used to analyze the interaction. FT-IR spectra were acquired for two cases: one with only TPEs and another with TPEs–Cu^2+^. The FT-IR spectra of the TPE–Cu^2+^ complex is suggested by the weakening or disappearance of hydroxide and/or amin stretching vibrations. This shift and weakening of hydroxide and amin peaks imply the involvement of the hydroxide and amine groups in the binding process (Figure S13).^36,40^

In this study, the novel TPEs exhibited on–off–on cascade recognition of copper and glutathione in aqueous solutions. We created two new compounds with similar structures, combining phenyl or naphthol units. Interestingly, the side groups attached to the main TPE structure did not affect attachment morphology or the amount of binding (LOD, *K*_a_, LOQ, etc.). In simpler terms, the side groups made no important difference besides creating novel TPEs. The performance of these sensors was evaluated in comparison to previously reported Cu^2+^ and GSH sensors, particularly in terms of *K*_a_ and LOD values. The results presented in Table 1 demonstrate that the sensors developed in this study offer comparable performance to those reported in the literature, making them promising candidates for copper and glutathione sensing applications.^36,40,43−52^

**Table 1:** Comparison of Some Cu^2+^ and GSH Selective Chemosensors

ref	sensing ions	binding constant (*K*_a_)	LOD
(36)	Cu^2+^	3.0 × 10^6^ M^–1^	15.7 μM
(40)	Cu^2+^, Hg^2+^	3.7 × 10^2^ M^–1/2^	2.42 μM
(43)	Cu^2+^, CN^–^	1.0 × 10^10^ M^–2^	900 nM
(44)	Cu^2+^, F^–^	2.5 × 10^3^ M^–1^	250 nM
(45)	Cu^2+^	5.9 × 10^4^ M^–1^	1.60 μM
(46)	Cu^2+^	2.8 × 10^4^ M^–1^	10.0 μM
(47)	Cu^2+^	1.1 × 10^3^ M^–1^	7.60 μM
(48)	Cu^2+^	4.0 × 10^9^ M^–2^	1.50 μM
(49)	Cu^2+^	1.5 × 10^5^ M^–1^	2.00 μM
(50)	GSH	ND	4.30 μM
(51)	GSH	ND	30.0 nM
(52)	GSH	ND	37.0 nM
this study	Cu^2+^ GSH	1.78 × 10^5^ M^–1/2^	597 nM
		ND	544 nM

**Figure 5:**
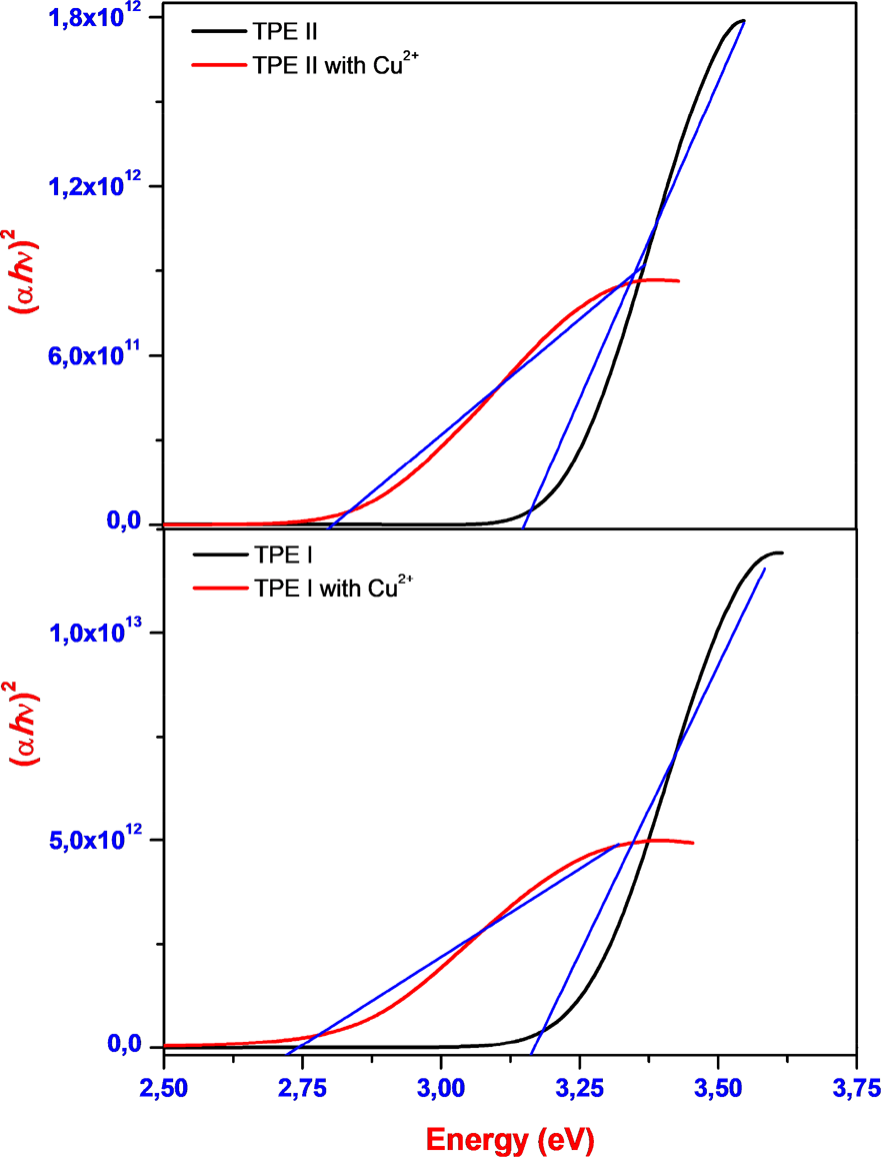
Band gap energies of TPE I/TPE I–Cu^2+^ and TPE II/TPE II–Cu^2+^.

Subsequent to the experimental UV–vis studies, the band gap energy (*E*_g_) values of TPEs and TPEs–Cu^2+^ were also determined experimentally (Figure 5 and S14). To accomplish this, the absorption coefficient (α) was initially calculated using the formula 4, where *d* represents the film thickness and *T* represents the percent optical transmittance value.
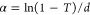
4

Subsequently, the *E*_g_ for TPEs and TPEs-Cu^2+^ was determined using the formula 5, where (*hν*) represents the photon energy and *K* is a material constant.^53^
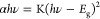
5

Furthermore, the *E*_g_ can be correlated with electrical conductivity and kinetic stability. An organic molecule with a wider HOMO–LUMO energy gap exhibits higher chemical hardness and stability, while a molecule with a narrower HOMO–LUMO energy gap demonstrates greater chemical reactivity. In this context, the experimental *E*_g_ values of **TPE I**/TPE I–Cu^2+^ and **TPE II/**TPE II–Cu^2+^ were calculated to be 3.18/2.72 eV and 3.10/2.78 eV, respectively (Figure 5). Moreover, on the basis of the experimental *E*_g_ values and the obtained absorbance measurements, it can be said that TPEs hold promise for photovoltaic applications. In addition to AIE and energy-band gap studies, the fluorescence quantum yield (Φ) was determined utilizing the standard proportional method.^54,55^ To establish the quantum yield of the TPEs in water, the quantum yield of rhodamine 101 (R101) in ethanol was normalized to (λ_ex_ = 500 nm, Φ_R_ = 1). The calculation of fluorescence quantum yields was conducted using the general eq 6 outlined below:
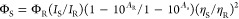
6

Here, Φ_S_ and Φ_R_ symbolize the quantum efficiency of the sample and the R101 reference (where Φ_R_ = 1). *I*_S_ and *I*_R_ represent the integrated emission intensity of the corrected spectrum, while *A*_S_ and *A*_R_ stand for the absorption values at excitation wavelengths for the sample and reference. η_S_ and η_R_ denote the refractive indexes of the solvents utilized for the sample and reference. In this context, the quantum yields of **TPE I** and **TPE II** were assessed at λ_ex_ = 370 nm, yielding 0.651 and 0.325, respectively (Figure S15).

**Figure 6:**
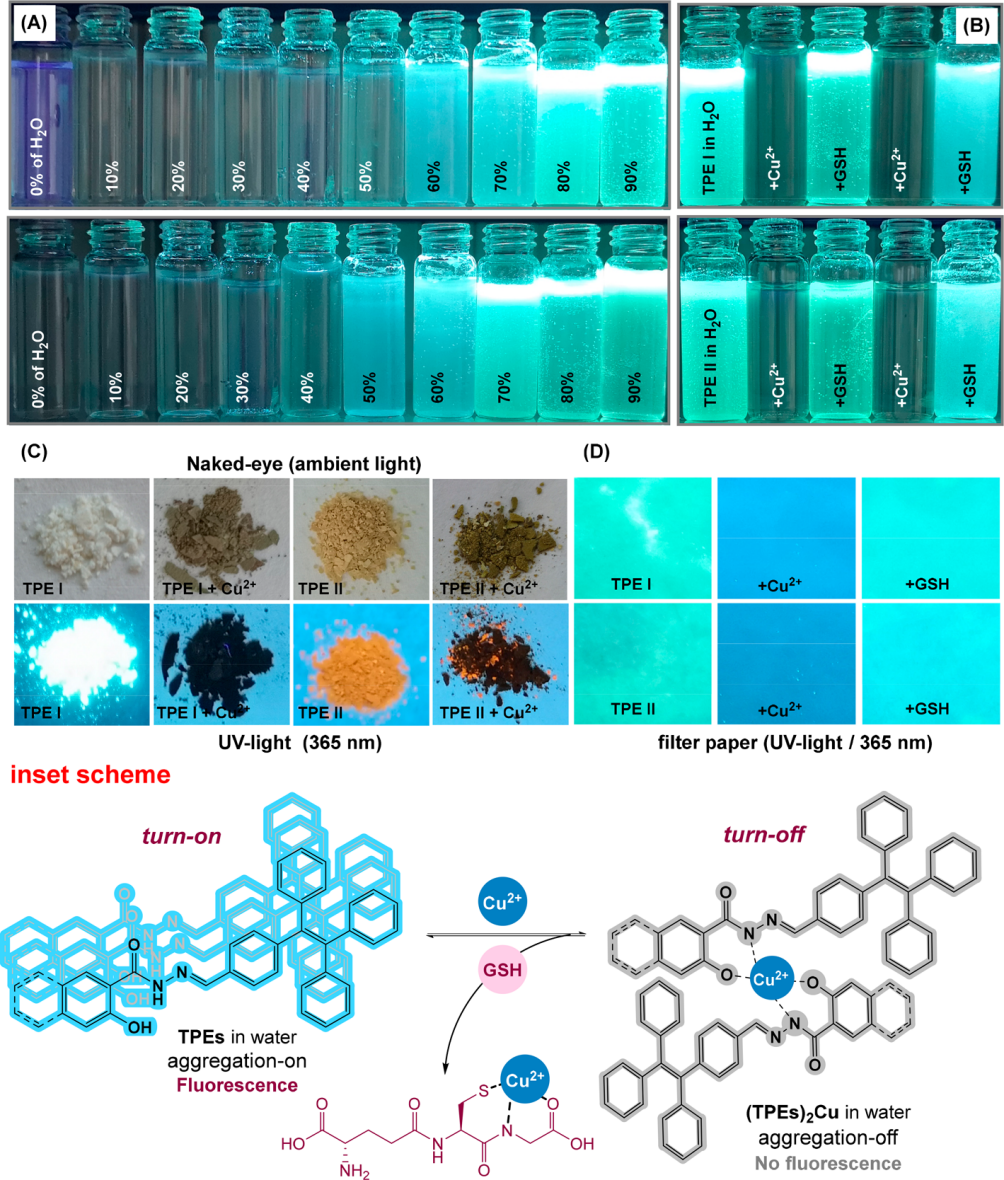
(A) The photographic images of TPEs in the various H_2_O ratios, (B) the color changes of TPEs upon alternate addition of Cu^2+^ and GSH for two cycles under UV-light, (C) the photographs of the solid-state of TPEs and TPEs-Cu^2+^ under ambient or UV- light, and (D) the color changes on test paper of TPEs upon alternate addition of Cu^2+^ and GSH for two cycles under UV light. Inset scheme: Proposed mechanism and structures of TPEs and GSH.

Colorimetric and filter paper studies are useful tools for evaluating sensor candidates, complementing instrumented measurements. For this purpose, we investigated our sensor candidate with these techniques, using real water samples. First, AIE studies demonstrated increased TPEs emissions with higher water content. This AIE behavior likely results from reduced photoinduced electron transfer as TPEs disaggregate in water due to their nucleophilic nitrogen (N) and oxygen (O) groups (Figure 6A).^56^ Reversible binding was confirmed by alternating Cu^2+^ and GSH additions, which respectively quenched and restored fluorescence (Figure 6B). TPEs samples, soft yellow and pale-orange solids, exhibit distinct emissions from TPEs–Cu^2+^ complexes, likely as a result of copper interfering with the π–π* transitions of the TPE moiety (Figure 6C).^33^ Finally, we explored practical applications using filter paper strips dipped in TPEs solutions. Dipping these strips in solutions containing both copper and GSH resulted in rapid, visible color changes under UV light (Figure 6D). This simple method offers a convenient and affordable way to visually detect copper and GSH, highlighting the potential of TPEs for developing user-friendly on–off–on sensors for these analytes. Overall, the interaction mechanism and the significance of water in this process can be explained like that. Namely, studies on AIE show that TPEs clump together (aggregate) in water, which makes them emit light (fluorescence). However, adding copper ions disrupts this aggregation, preventing TPEs from clumping and causing a decrease in fluorescence intensity. Interestingly, when GSH is introduced, it binds to the copper ions, effectively removing them. This allows the TPEs to aggregate again in the presence of water, and their fluorescence is restored (Figure 6, inset scheme).

## 3 Conclusions

In summary, we successfully synthesized and characterized two novel **TPEsI** and **II**, and explored their photochemical properties. We evaluated their interactions with various ions in diverse solvents, finding that both TPEs specifically exhibited a turn-off sensing response toward Cu^2+^. Interestingly, the TPEs–Cu^2+^ system displayed a unique turn-on fluorescence response upon subsequent exposure to GSH, indicating reversible sensing capabilities and the potential for TPEs reuse. The calculated LOD, LOQ, and *K*_a_ values for both TPE I–Cu^2+^ and TPE II–Cu^2+^ were 747/597 nM, 2.26/1.81 μM, and 2.46 × 10^5^/1.78 × 10^5^ M^1/2^, respectively. Furthermore, the LOD and LOQ values for TPE II–Cu^2+^–GSH were calculated as 547 nm and 1.65 μM, respectively. Moreover, the quantum yields of **TPE I** and **TPE II** were also assessed at λ_ex_ = 370 nm, yielding 0.651 and 0.325, respectively. In addition, the investigation also employed visual analysis to assess the color alterations of TPEs on filter paper and in real water samples. This approach proved successful in systems containing copper and GSH. Overall, this work demonstrates effective procedures for synthesizing TPEs and their application in detecting both copper and GSH in aqua solutions.

## 4 Experimental Section

### 4.1 Synthesis of TPEs

The novel tetraphenylethene derivatives (TPEs), **TPE I** and **TPE II**, were synthesized from readily available 4-(1,2,2-triphenylvinyl)benzaldehyde (TPE-CHO) through a two-step reaction (Scheme S1, Figures 1 and S2). First, TPE-CHO (500 mg, 1.39 mmol) was dissolved in ethanol (15 mL) and reacted with either 2-hydroxybenzohydrazide (211 mg, 1.39 mmol) for **TPE I** or 3-hydroxy-2-naphthohydrazide (281 mg, 1.39 mmol) for **TPE II**. The reaction mixtures were then heated under reflux overnight, cooled to room temperature, and the crude products were filtered and recrystallized from ethanol. This afforded the desired **TPE I** and **TPE II** as yellow solids in 85% and 72% yields, respectively. Detailed experimental procedures and spectroscopic data are provided in the SI. **TPE I**: ^1^H NMR (400 MHz, DMSO-d6) δ 11.87 (bs, OH, 1H), 11.83 (bs, NH, 1H), 8.35 (s, N=CH, 1H), 7.88 (d, *J* = 8.1 Hz, =CH, 1H), 7.50 (m, A part of AB system, =CH, 2H), 7.43 (t, *J* = 8.1 Hz, =CH, 1H), 7.11–7.16 (m, =CH, 9H), 7.05 (m, B part of AB system, =CH, 2H), 6.93–7.00 (m, =CH, 8H); ^13^C NMR (100 MHz, CDCl_3_) δ 165.17, 159.51, 148.76, 145.76, 143.44 (2C), 143.26, 141.86, 140.48, 134.33, 132.70, 131.66, 131.19, 131.17, 131.12, 129.02, 128.43 (2C), 128.33, 127.28, 127.22 (3C), 119.43, 117.43, 116.31 (Figure S3). **TPE II**: ^1^H NMR (400 MHz, DMSO-d6) δ 11.97 (bs, OH, 1H), 11.33 (bs, NH, 1H), 8.44 (s, N=CH, 1H), 8.36 (s, =CH, 1H), 7.91 (d, *J* = 8.2 Hz, =CH, 1H), 7.76 (d, *J* = 8.2 Hz, =CH, 1H), 7.52 (m, A part of AB system, =CH, 2H), 7.32–7.38 (m, =CH, 2H), 7.11–7.16 (m, =CH, 10H), 7.06 (m, B part of AB system, =CH, 2H), 6.98–7.01 (m, =CH, 6H); ^13^C NMR (100 MHz, CDCl_3_) δ 164.21, 154.59, 148.63, 145.76, 143.45 (2C), 143.26, 141.88, 140.51, 136.34, 132.76, 131.66, 131.19 (2C), 131.16, 131.11, 130.74, 129.15, 128.42, 128.32 (2C), 127.28, 127.22 (3C), 127.17, 126.32, 124.28, 120.69, 111.07 (Figure S4).

### 4.2 Procedures of Photophysical Measurement

UV–vis and fluorescence spectra of **TPE I** and **TPE II** were recorded in the presence of various ions. The measurements were performed in EtOH/HEPES at room temperature, adding one equivalent of each ion at a time. A separate fluorescence titration study was conducted for **TPE I** and **TPE II** with CuCl_2_. This involved adding different concentrations of CuCl_2_ solution to a solution of **TPE I** or **TPE II** in EtOH/HEPES (v/v:0.1/9.9) or real water samples. All measurements were repeated at least twice to ensure consistency. Additionally, Job’s plot analysis was used to determine the stoichiometry of the interaction, and the LOD, the LOQ, and the *K*_a_ values were calculated using appropriate formulas. The detailed experimental procedures can be found in the supplementary data.

## Supporting Information Available

General experimental methods and characterizations of the materials (PDF)
