# Supplementary material for: Synthesis of Phenol-Hydrazide-Appended Tetraphenylethenes as Novel On–Off–On Cascade Sensors of Copper and Glutathione
Source: ACS Omega. 2024 Jun 7;9(24):26257–66. doi: 10.1021/acsomega.4c02043 (PMC11191134; doi:10.1021/acsomega.4c02043)
Supplement: Supplementary file 1 — ao4c02043_si_001.pdf [file ao4c02043_si_001.pdf]

Supplementary Data for

Synthesis of Phenol-Hydrazide-Appended Tetraphenylethenes as Novel On-Off-On Cascade Sensors of Copper and Glutathione

Sinan Bayindir[a]* and Sebiha Akar[b]

[a]Department of Chemistry, Faculty of Sciences and Arts, Bingöl University, Bingöl, 12000, Turkey
[b]Department of Chemistry, Graduate School of Natural and Applied Sciences, Bingöl University, Bingöl, 12000,Türkiye

*Corresponding author:

E-mail address: *sbayindir@bingol.edu.tr*

General methods

All chemicals, reagents, and solvents were commercially available from Sigma-Aldrich or Merck. Melting point was determined on a Buchi 539 capillary melting apparatus and is uncorrected. Infrared spectra were recorded on a Mattson 1000 FT-IR spectrophotometer. ^{1}H NMR and ^{13}C NMR spectra were recorded on a 400 (100)-MHz Varian and Bruker spectrometer and are reported in terms of chemical shift (δ, ppm) with $SiMe_4$ as an internal standard. Data for ^{1}H NMR are recorded as follows: chemical shift (δ, ppm), multiplicity (s: singlet, d: doublet, t: triplet, q: quartet, p: pentet, m: multiplet, bs: broad singlet, bd: broad doublet, qd: quasi doublet) and coupling constant (s) in Hz, integration. Elemental analyses were carried out on a LECO CHNS-932 instrument. Column chromatography was carried out on silica gel 60 (230–400 mesh ASTM). The reaction progress was monitored by thin-layer chromatography (TLC) (0.25-mm-thick precoated silica plates: Merck Fertigplatten Kieselgel (60 F254)). UV-Vis absorption and fluorescence spectra of samples were recorded on a Shimadzu UV-3101PL UV-Vis-NIR spectrometer and Perkin–Elmer (Model LS 55) Fluorescence Spectrophotometer, respectively.

Synthesis of TPEs

*2-(4-Bromophenyl)ethene-1,1,2-triyl)tribenzene (***3***)*[1] *:* The n-butyllithium (n-BuLi, 2.23 mL, 5.95 mmol, in hexane) was added drop wise to a stirred solution of diphenylmethane (**2**; 1.00 g, 5.95 mmol) in dry THF (35 mL) at 0°C under nitrogen atmosphere, and the mixture was stirred for 2h at 0 °C. A solution of 4-bromobenzophenone (**1**; 1.30 g, 4.96 mmol) in THF (15 mL) was added to this mixture, and the reaction was stirred at room temperature for overnight. After completion of the reaction, the mixture was quenched by adding an aqueous solution of NH_4Cl, and then the mixture was extracted with CH_2Cl_2 (3×50 mL). The organic layers were combined and dried over anhydrous Na_2SO_4, and the solvent was evaporated to give a crude alcohol intermediate. The alcohol intermediate was dissolved in toluene (30 mL), and *p*-toluenesulfonic acid (*p*-TSA, 250 mg) was added to it and refluxed for 16 h. After the mixture was cooled to room temperature was evaporated on a rotary evaporator to give a crude residue which was purified by silica gel chromatography with hexane to give **3** (1.71 g, 83%) as white solid. ^{1}H NMR (400 MHz, $CDCl_3$) δ 7.25-7.21 (m, AA′ part of AA′BB′ system, =CH, 2H), 7.16-7.09 (m, =CH, 9H), 7.08-6.99 (m, =CH, 6H), 6.93-6.89 (m, BB′ part of AA′BB′ system, =CH, 2H). ^{13}C NMR (100 MHz, $CDCl_3$) δ 143.6, 143.5, 143.4, 142.9, 141.8, 139.9, 133.2, 131.5(3C), 131.1, 128.1, 128.0, 127.9, 126.9(2C), 126.8, 120.7 (Figure S1).

*4-(1,2,2-Triphenylvinyl)benzaldehyde (***TPE-CHO***)* [1]*:* To a stirred solution of **3** (500.0 mg, 1.22 mmol) in 25 mL anhydrous THF at -78 °C was added to n-butyllithium (1.17 mL, 2.92 mmol in hexane) drop wise under nitrogen atmosphere, and the reaction mixture was stirred for 2h at this temperature. *N*-formylpiperidine (NFP, 206 mg, 1.83 mmol) was injected in one portion. The solution was warmed to room temperature gradually and stirred overnight. The reaction was

quenched by adding 50 mL of 2 M HCl solution. The organic layer was separated, and the aqueous layer was extracted with diethyl ether (3×50 mL). The organic layers were combined and dried over anhydrous Na_2SO_4. After the solvent was removed under reduced pressure, the residue was purified by silica gel column chromatography using petroleum Et_2O/EtOAc (99:1) as eluent to give the desired product **TPE-CHO** (355 mg, 81%) as a yellow solid. ^1H NMR (400 MHz, $CDCl_3$) δ 9.91 (s, CHO, 1H), 7.65-7.61 (m, AA′ part of AA′BB′ system, =CH, 2H), 7.23-7.19 (m, BB′ part of AA′BB′ system, =CH, 2H), 7.16-7.10 (m, =CH, 9H), 7.08-7.00 (m, =CH, 6 H). ^{13}C NMR (100 MHz, $CDCl_3$) δ 192.1, 150.8, 143.3 (2C), 143.2, 143.1, 140.0, 134.5, 132.2, 131.5(2C), 129.4, 128.1(2C), 128.0, 127.3, 127.1(2C) (Figure S2).

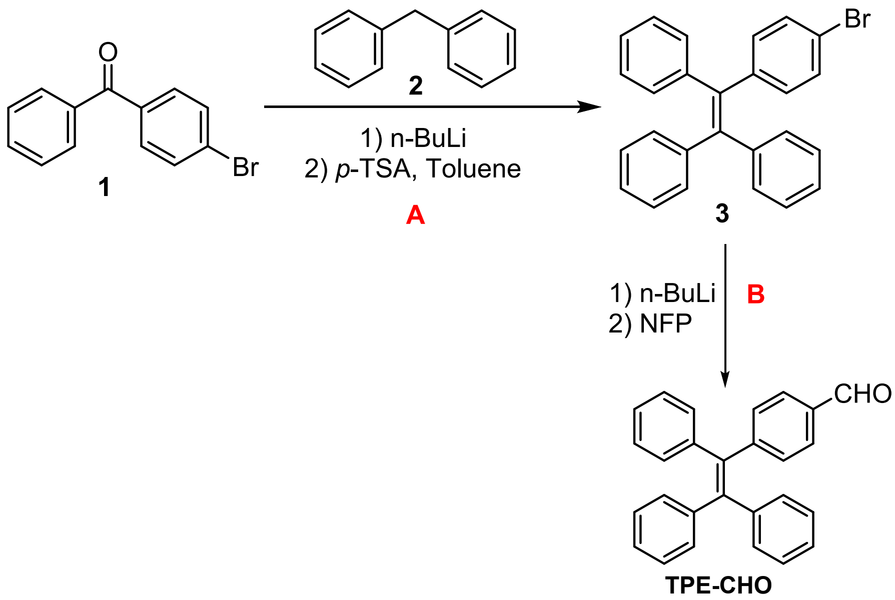

Scheme S1. The synthesis strategy of **TPE-CHO**

The novel tetraphenylethene derivatives (TPEs): **TPE I** and **TPE II** were synthesized from readily available 4-(1,2,2-triphenylvinyl)benzaldehyde (TPE-CHO) through a two-step reaction (Scheme S1, Fig. 1 and S2). First, TPE-CHO (500 mg, 1.39 mmol) was dissolved in ethanol (15 mL) and reacted with either 2-hydroxybenzohydrazide (211 mg, 1.39 mmol) for **TPE I** or 3-hydroxy-2-naphthohydrazide (281 mg, 1.39 mmol) for **TPE II**. The reaction mixtures were then heated under reflux overnight, cooled to room temperature, and the crude products were filtered and recrystallized from ethanol. This afforded the desired **TPE I** and **TPE II** as yellow solids in 85% and 72% yields, respectively. Detailed experimental procedures and spectroscopic data are provided in the supplementary information. **TPE I:** ^1H NMR (400 MHz, DMSO-d6) δ 11.87 (bs, OH, 1H), 11.83 (bs, NH, 1H), 8.35 (s, N=CH, 1H), 7.88 (d, J = 8.1 Hz, =CH, 1H), 7.50 (m, A part of AB system, =CH, 2H), 7.43 (t, J = 8.1 Hz, =CH, 1H), 7.11-7.16 (m, =CH, 9H), 7.05 (m, B part of AB system, =CH, 2H), 6.93-7.00 (m, =CH, 8H); ^{13}C NMR (100 MHz, $CDCl_3$) δ 165.17, 159.51, 148.76,

145.76, 143.44 (2C), 143.26, 141.86, 140.48, 134.33, 132.70, 131.66, 131.19, 131.17, 131.12, 129.02, 128.43 (2C), 128.33, 127.28, 127.22 (3C), 119.43, 117.43, 116.31; ESI-MS $C_{34}H_{26}N_2O_2$ (m/z) $[M]^+$ = 494.20 (Figure S3). **TPE II:** ^{1}H NMR (400 MHz, DMSO-d6) δ 11.97 (bs, OH, 1H), 11.33 (bs, NH, 1H), 8.44 (s, N=CH, 1H), 8.36 (s, =CH, 1H), 7.91 (d, *J* = 8.2 Hz, =CH, 1H), 7.76 (d, *J* = 8.2 Hz, =CH, 1H), 7.52 (m, A part of AB system, =CH, 2H), 7.32-7.38 (m, =CH, 2H), 7.11-7.16 (m, =CH, 10H), 7.06 (m, B part of AB system, =CH, 2H), 6.98-7.01 (m, =CH, 6H); ^{13}C NMR (100 MHz, $CDCl_3$) δ 164.21, 154.59, 148.63, 145.76, 143.45 (2C), 143.26, 141.88, 140.51, 136.34, 132.76, 131.66, 131.19 (2C), 131.16, 131.11, 130.74, 129.15, 128.42, 128.32 (2C), 127.28, 127.22 (3C), 127.17, 126.32, 124.28, 120.69, 111.07; ESI-MS $C_{38}H_{28}N_2O_2$ (m/z) $[M]^+$ = 544.22 (Figure S4).

UV-Vis and fluorescence studies of TPEs with various cations: The solution of **TPEs** (1×10^{-2} M) and cations (chloride salt, 1×10^{-2} M) were prepared in EtOH and H_2O, respectively. A solution of TPEs (10 μM) was placed in a quartz cell and the UV-Vis and fluorescence spectrums were recorded in EtOH/HEPES (v/v:1/9). After introduction of the solution of cations (1 equiv.), the changes in absorbance intensity were recorded at room temperature each time.

UV-Vis and fluorescence titration of TPEs with $CuCl_2$: The solution of probe TPES (1×10^{-2} M) and $CuCl_2$ (1×10^{-2} M) were prepared in EtOH and H_2O, respectively. The concentration of probe TPEs used in the experiments was 10 μM. The UV-Vis and fluorescence titration spectras were recorded by adding corresponding concentration of $CuCl_2$ to a solution of TPEs in EtOH/HEPES (v/v:1/9). Each titration was repeated at least twice until consistent values were obtained.

Job's plot measurement: Probe TPEs was dissolved in EtOH/HEPES (v/v:1/9) to make the concentration of 1×10^{-2} M. 5.00, 4.50, 4.00, 3.50, 3.00, 2.50, 2.00, 1.50, 1.00, 0.50 and 0.0 mL of the ligand solution were taken and transferred to vials. $CuCl_2$ was dissolved in H_2O to make the concentration of 1×10^{-2} M. 0.0, 0.50, 1.00, 1.50, 2.00, 2.50, 3.00, 3.50, 4.00, 4.50, and 5 mL of the $CuCl_2$ solution were added to each ligand solution. Each vial had a total volume of 5 mL. After shaking the vials for a few seconds, absorbance spectras were taken at room temperature.

Determination of detection limit: The fluorescence measurements were taken for each solution containing Cu^{2+} ions. The detection limit for Cu^{2+} was calculated based on the fluorescence titration. For this purpose, $3\,s/k$ equation was used. Where s the standard deviation of blank, k is the slope of the fit line in fluorescence titration experiment or in absorbance titration experiment.

Determination of Association Constant: Association constant was calculated according to the Benesi-Hildebrand equation by fluorescent method. Association Constant (K_a) was calculated following the equation stated below:

$$\frac{1}{F-F_0}=\frac{1}{\{K_a(F_{max}-F_0)[M^{x+}]^n\}}+\frac{1}{F_{max}-F_0}$$

Here, F_o is the fluorescence of receptor in the absence of metal ion, F is the fluorescence recorded in the presence of added metal ion, F_{max} is fluorescence in presence of added $[M^{x+}]$ max and K_a is the association constant, where $[M^{x+}]$ is $[Cu^{2+}]$. n is the binding stoichiometry for receptor and metal ion. The association constant (K_a) could be determined from the slope of the straight line of the plot of $1/F_o$-F against $1/[Cu^{2+}]^{1/2}$.

The pH measurement: The effect of different pH environments (range of 2–12) was studied for the practical application of the TPEs (10 μM, in EtOH) in the absence and presence of Cu^{2+} (30 μM, in H_2O). For this purpose, the TPEs samples were prepared in ten different tubes, and copper ions dissolved in water were added. The pH values of samples were modulated by adding HCl or NaOH solution. pH values of the solution were monitored with a pH meter and/or pH stick.

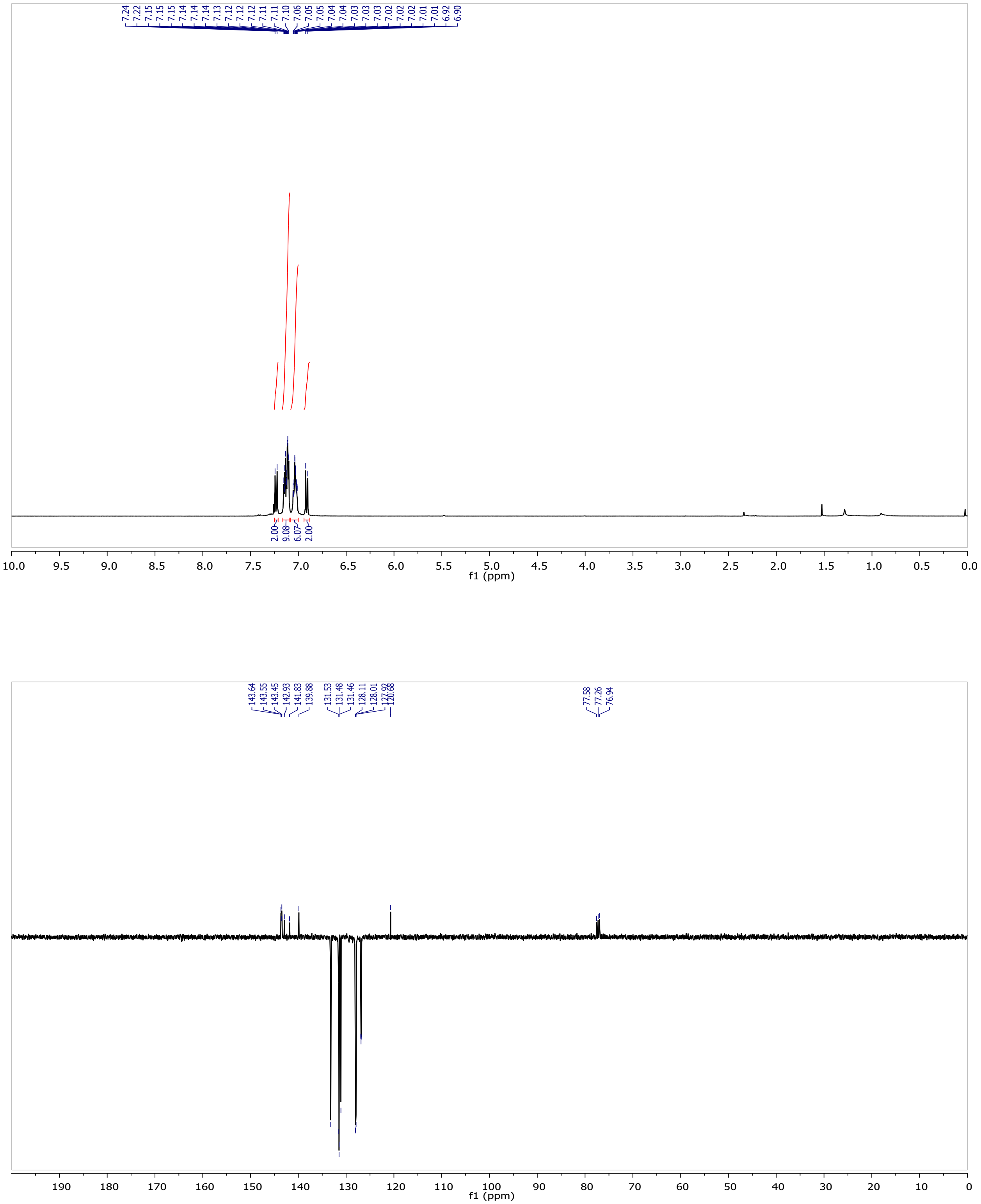

Figure S1. ^{1}H-NMR (400 MHz) and APT ^{13}C-NMR (100 MHz) spectrums of 3 in $CDCl_3$.

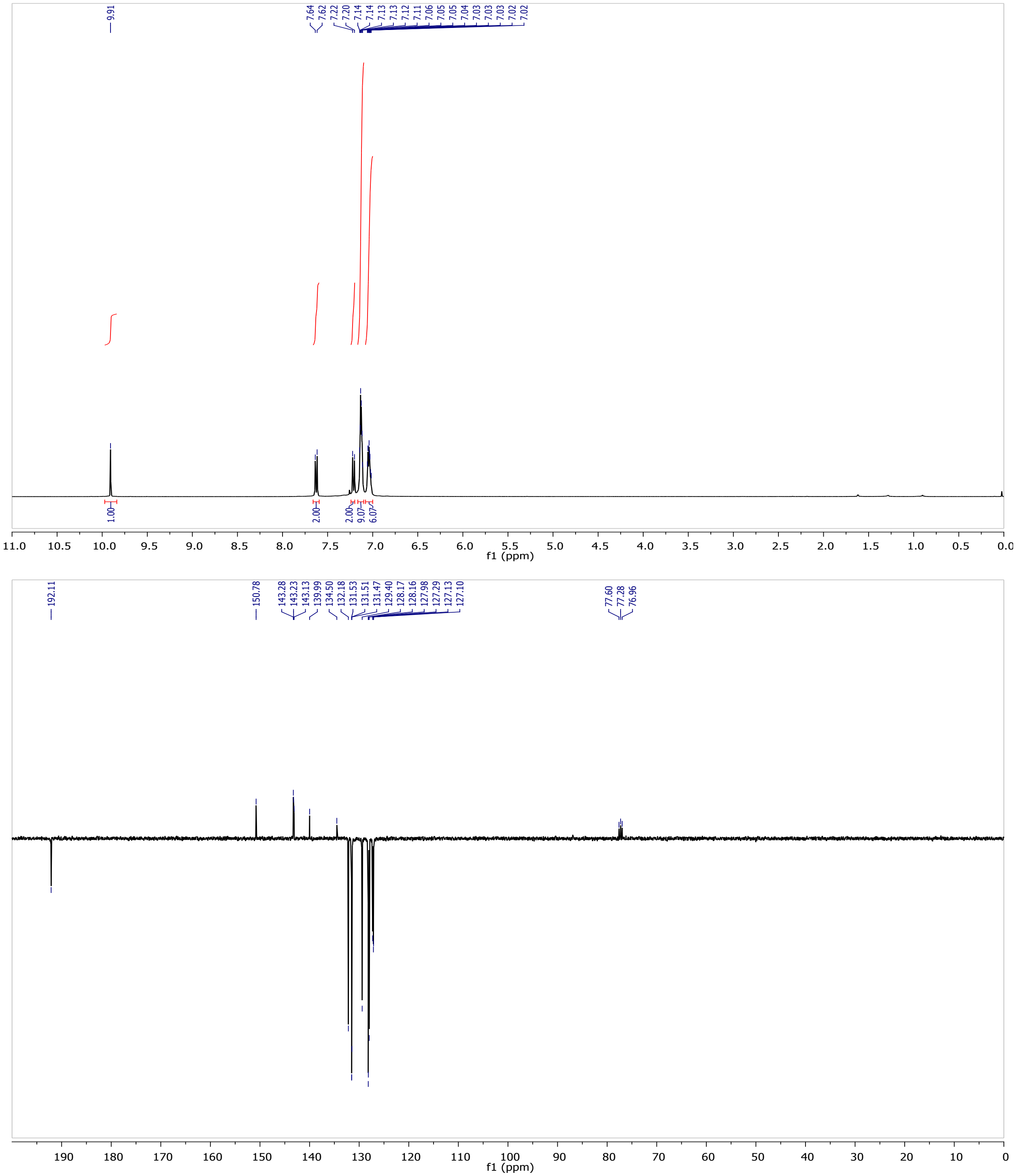

Figure S2. ^{1}H-NMR (400 MHz) and APT ^{13}C-NMR (100 MHz) spectrums of TPE-CHO in $CDCl_3$.

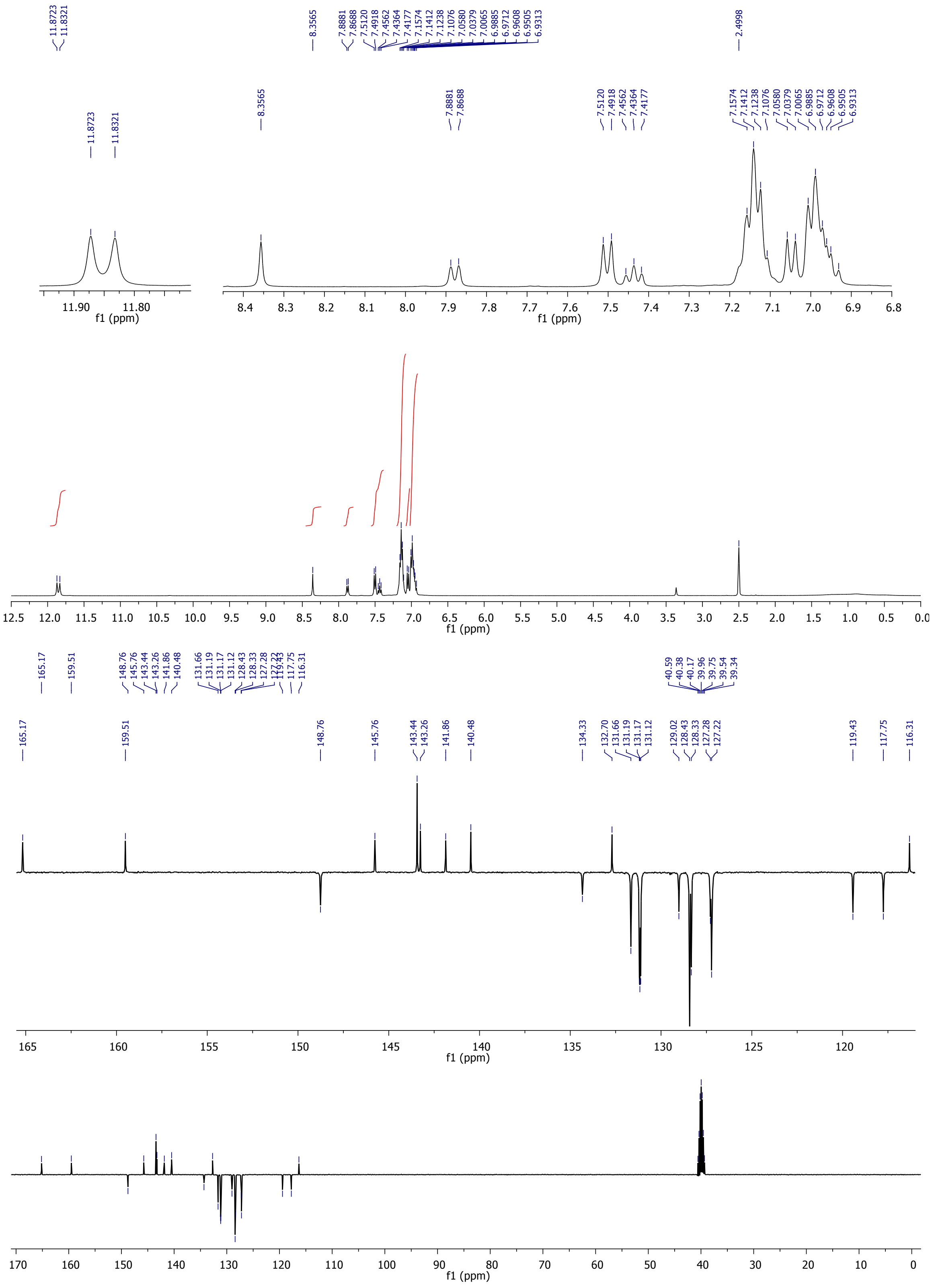

Figure S3. ^{1}H-NMR (400 MHz) and APT ^{13}C-NMR (100 MHz) spectrums of TPE I in DMSO-d6.

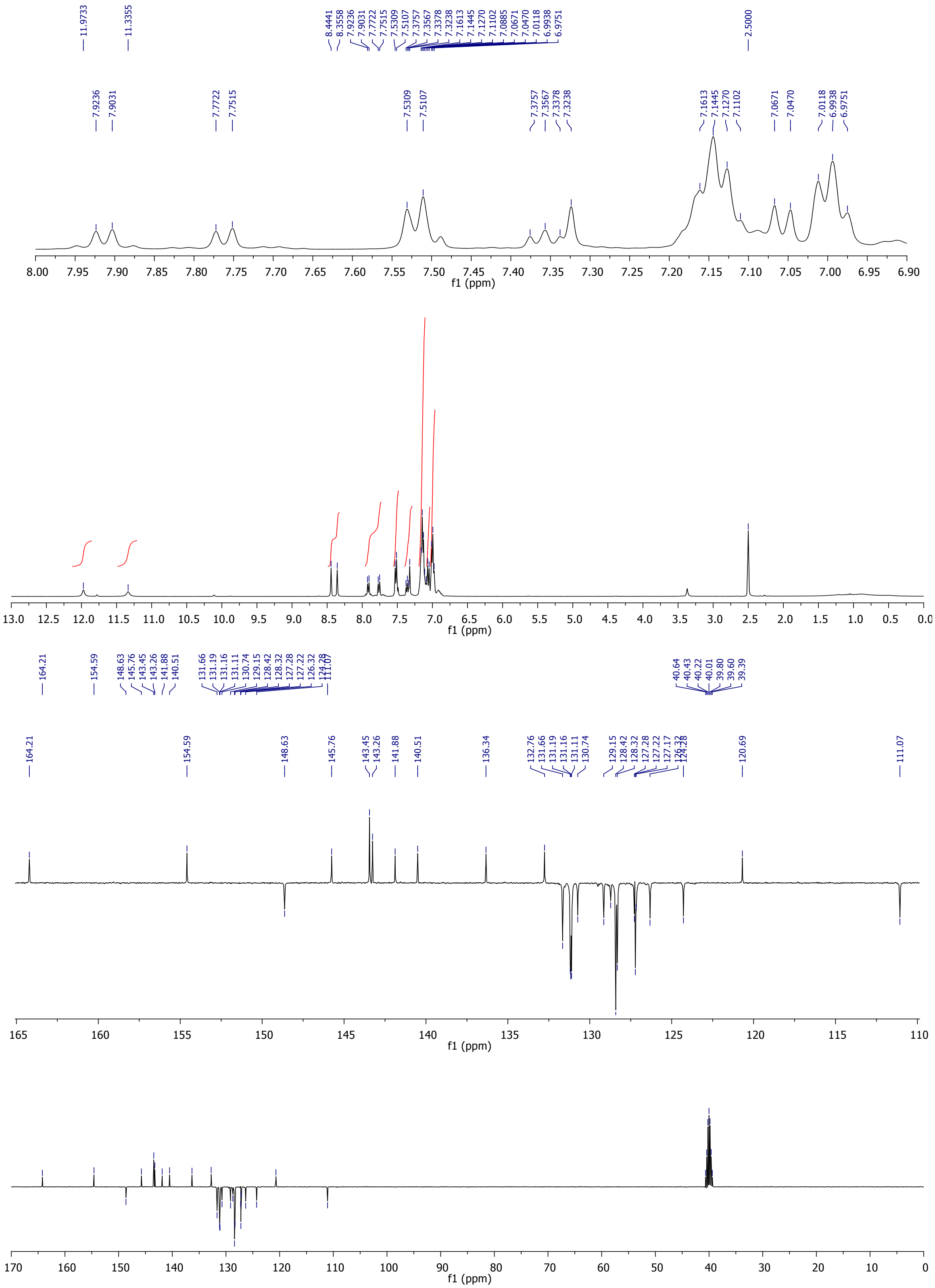

Figure S4. ¹H-NMR (400 MHz) and APT ¹³C-NMR (100 MHz) spectrums of TPE **II** in DMSO-d6.

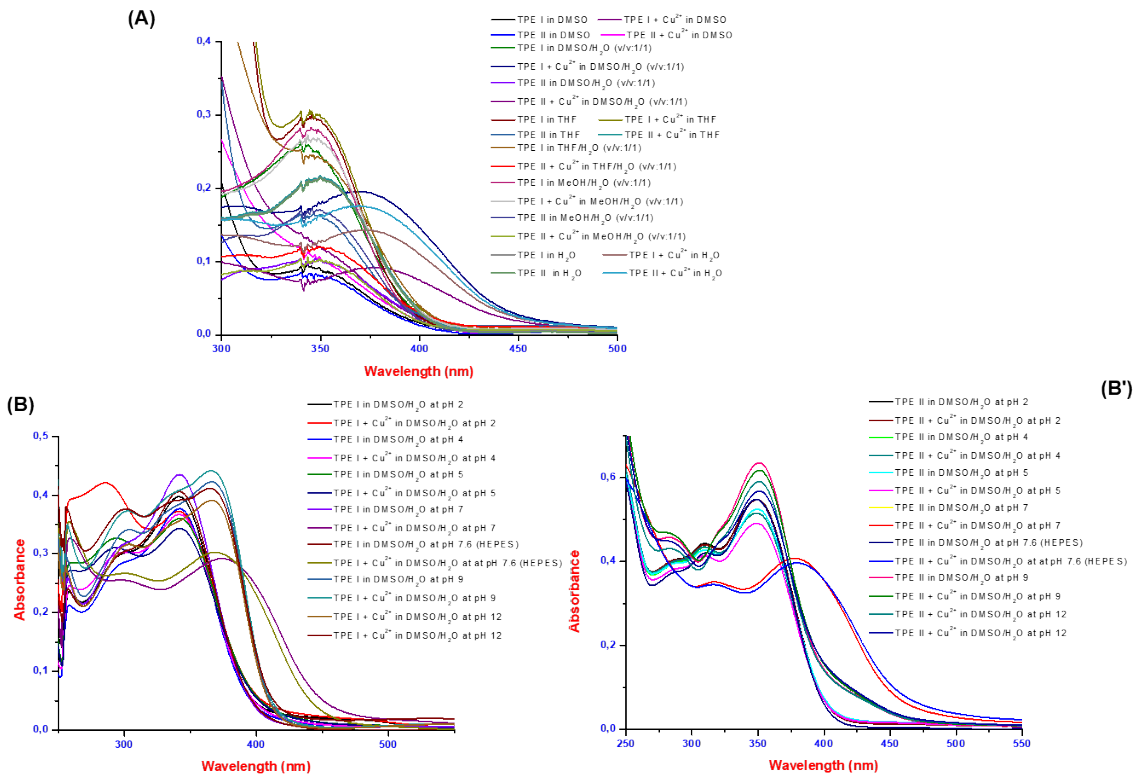

Figure S5. (**A**) Absorbance spectra of TPEs in variety solvent systems, and (**B** and **B'**) absorbance spectras of TPEs with [$CuCl_2$] at different pH (3–11) in EtOH/H_2O (v/v : 1/9), the pH was modulated by adding 75% HCl or NaOH solution.

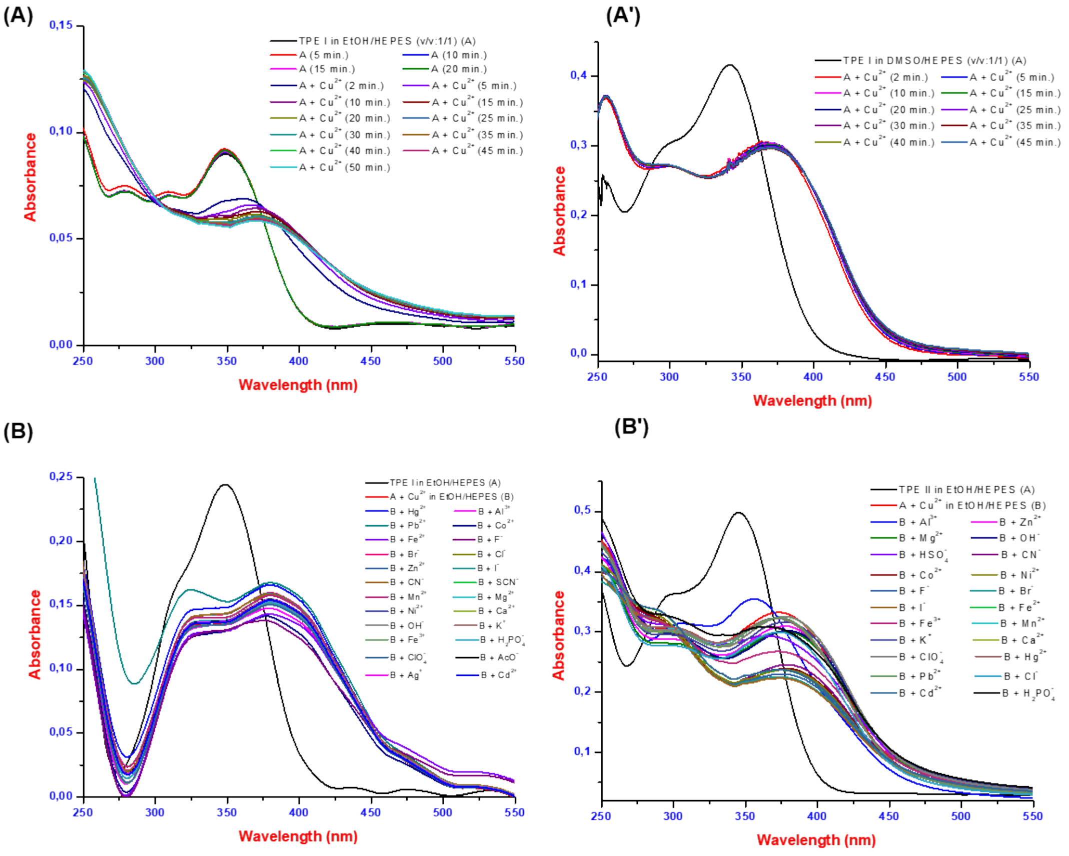

Figure S6. Absorbance enhancing profile of addition Cu^{2+} to TPEs in EtOH/H_2O (v/v-1/9) from 10 min to 60 min (A and A'), and absorbance spectras of TPEs in the presence of Cu^{2+} upon the addition of different metal ions in EtOH/H_2O (v/v-1/9) up to 1 equiv. (B and B')

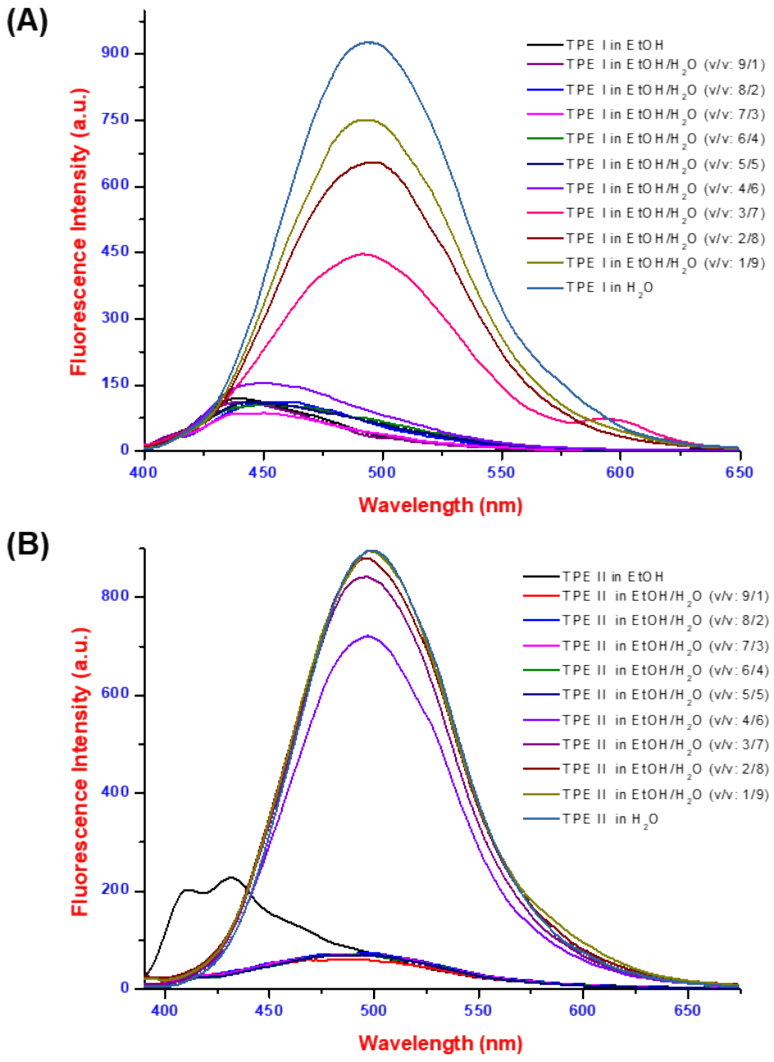

Figure S7. The AIE fluorescent response of TPE **I** (**A**) and TPE **II** (**B**) in different water ratio mixtures

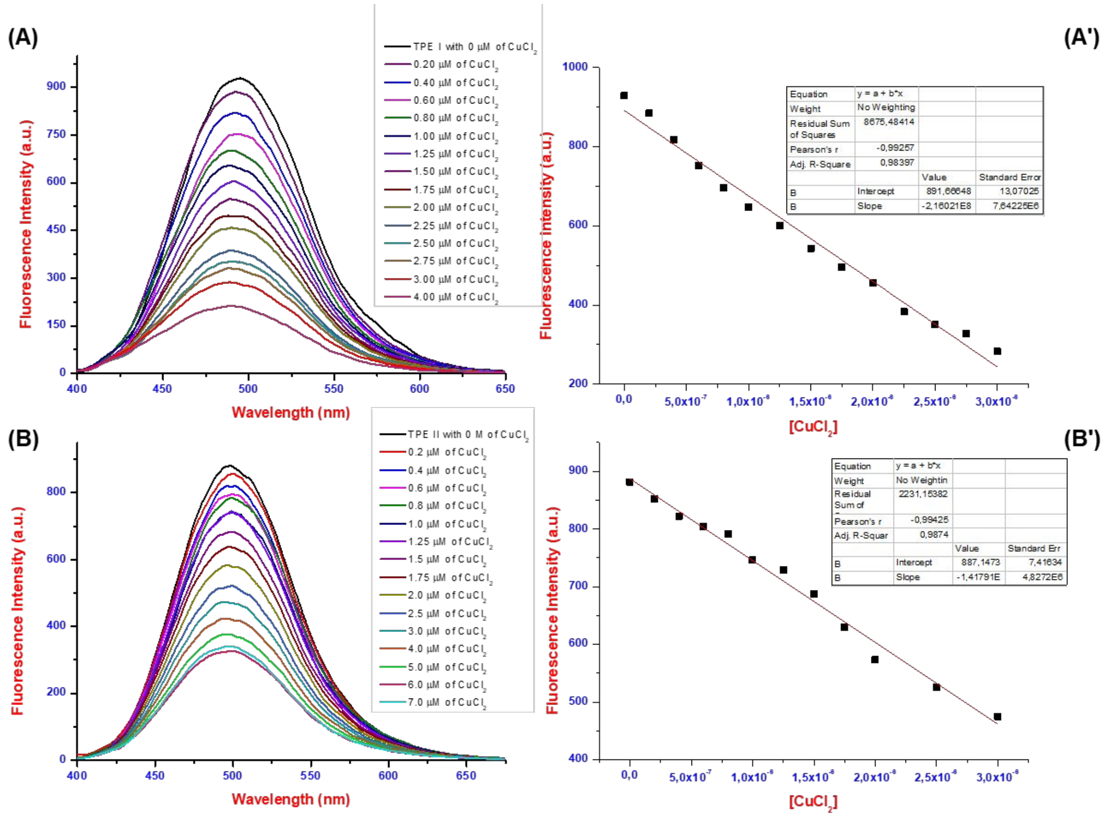

Figure S8. Fluorescence titration spectras of TPE **I** (**A**) or TPE **II** (**B'**) with $CuCl_2$ and change fluorescence intensity of TPE **I** (**A'**) or TPE **II** (**B'**) with the increasing concentration of Cu^{2+} in EtOH-H_2O (v/v-1:9).

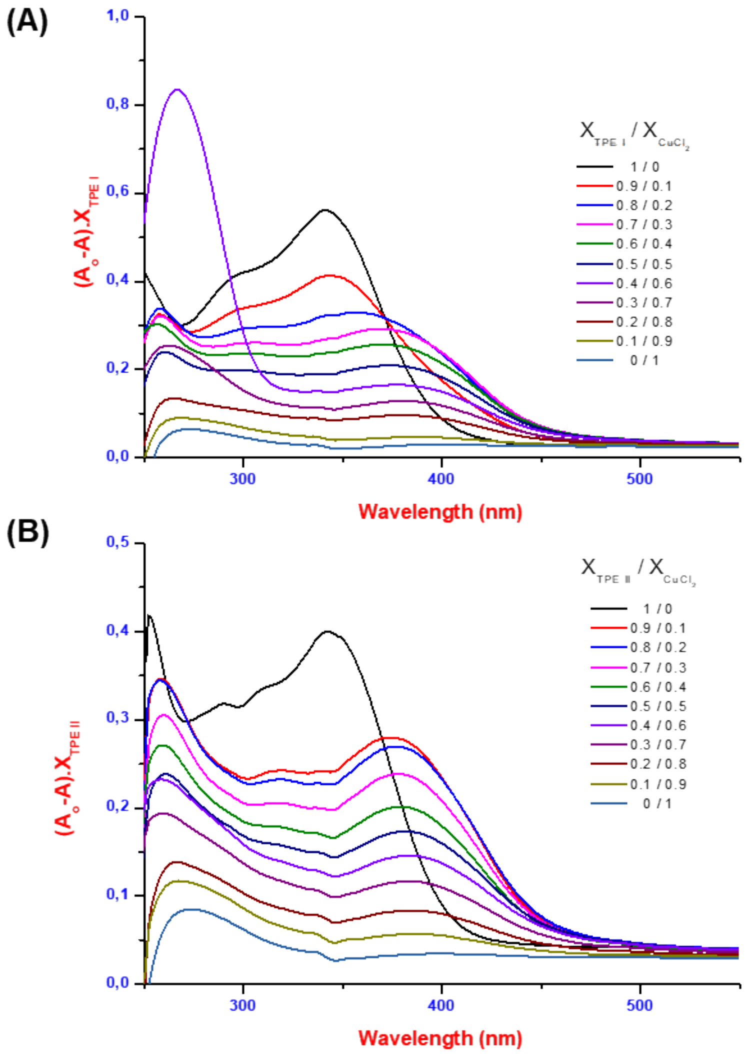

Figure S9. The Job plot absorbance spectras of TPE **I** (**A**) and TPE **II** (**B**) in EtOH/H_2O (v/v:1/9)

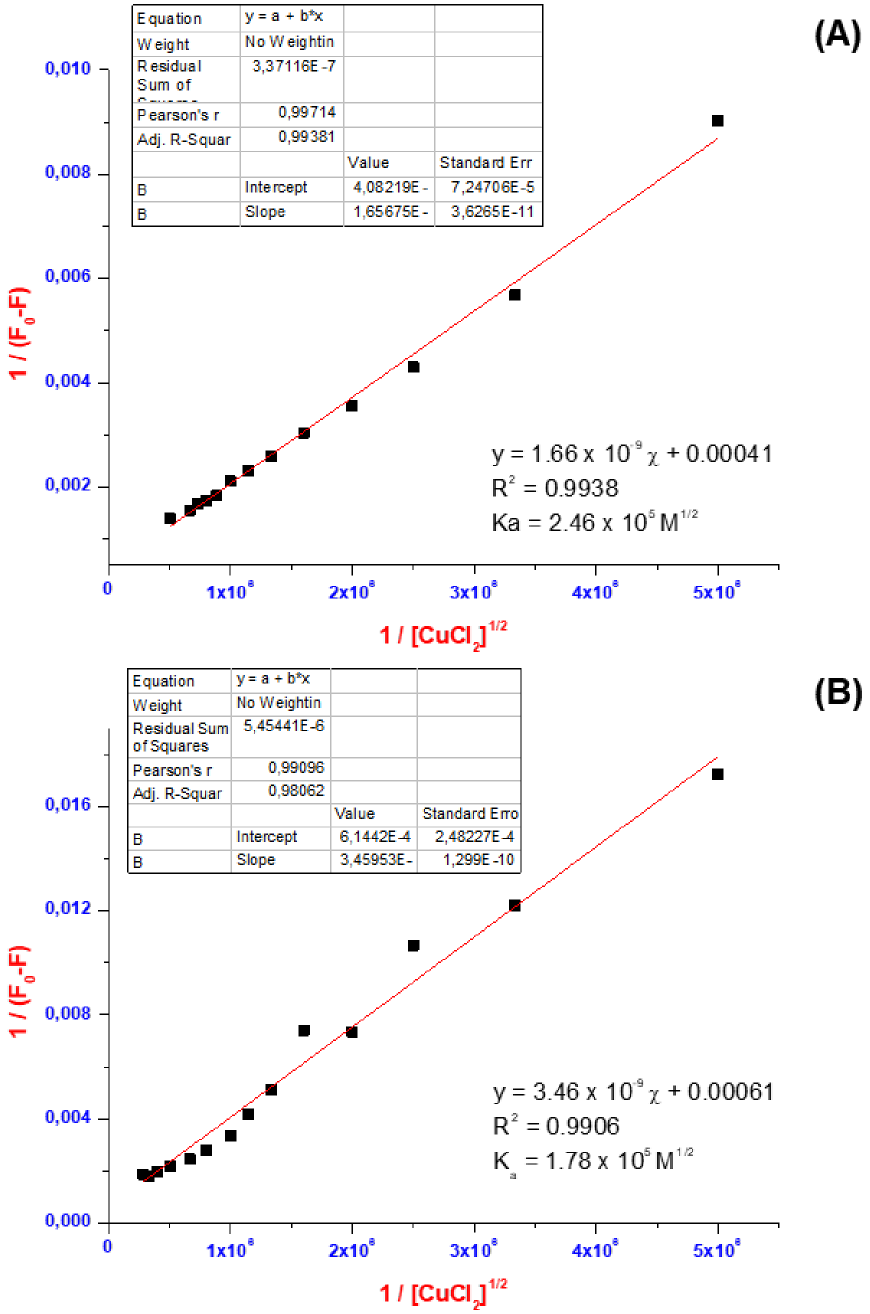

Figure S10. Benesi–Hildebrand plot based on a 1:2 association stoichiometry between TPE I (**A**) and TPE II (**B**) with Cu^{2+}

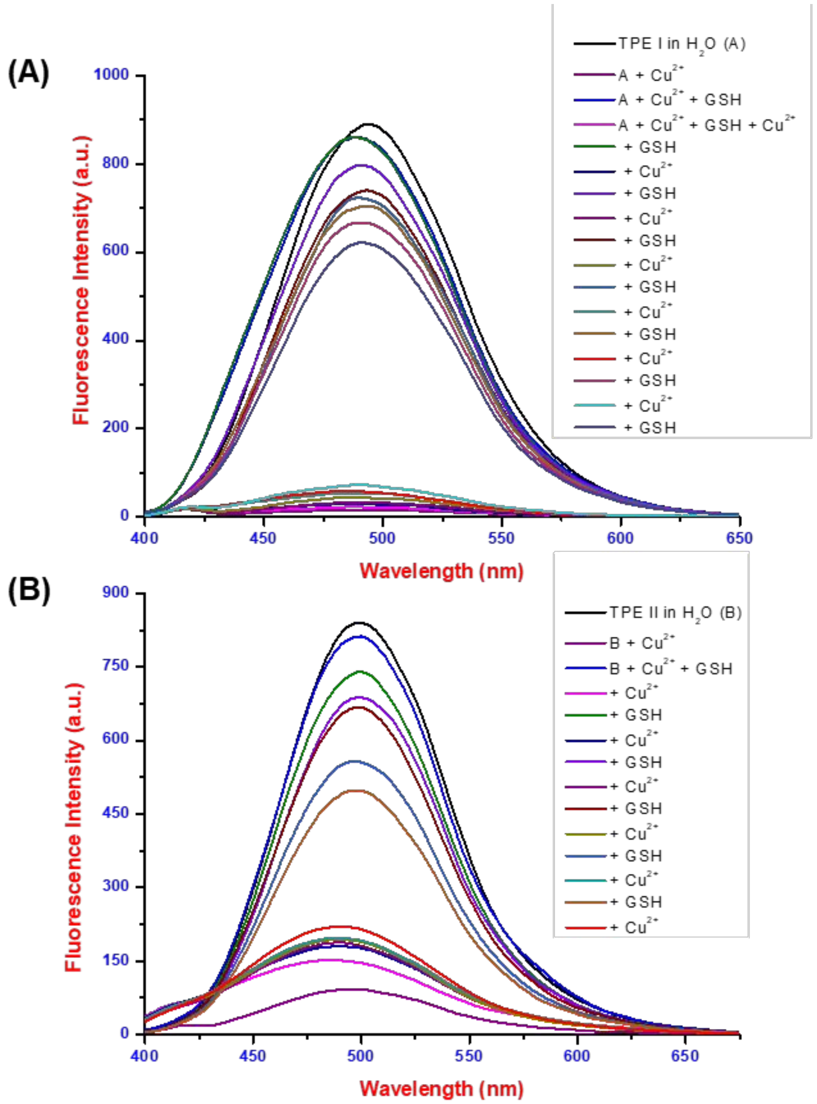

Figure S11. Reversible switching of the fluorescence spectrum of TPE **I** (**A**) and TPE **II** (**B**) with alternate addition of Cu^{2+} and GSH in EtOH-H_2O (v/v-1:9).

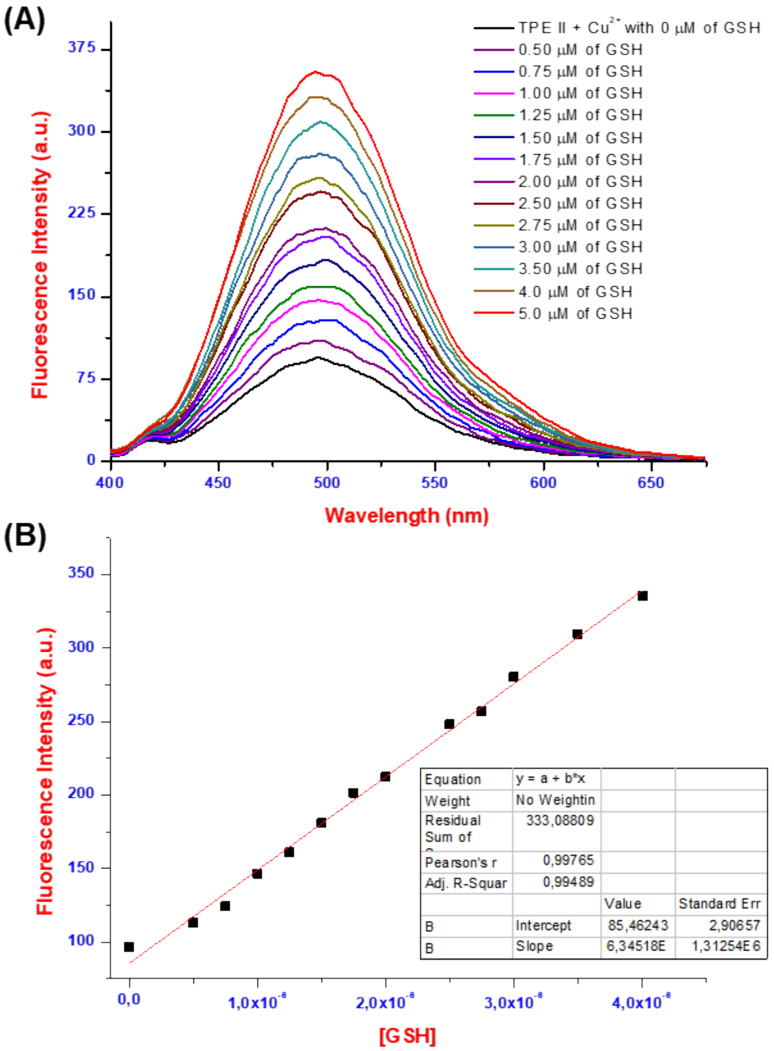

Figure S12. (**A**) The fluorescence titration spectra of TPE II-Cu^{2+} in EtOH/H_2O (v/v-1/9) in the presence of GSH, and (**B**) the change fluorescence intensity of TPE II-Cu^{2+} with the increasing concentration of GSH in EtOH/H_2O (v/v-1/9).

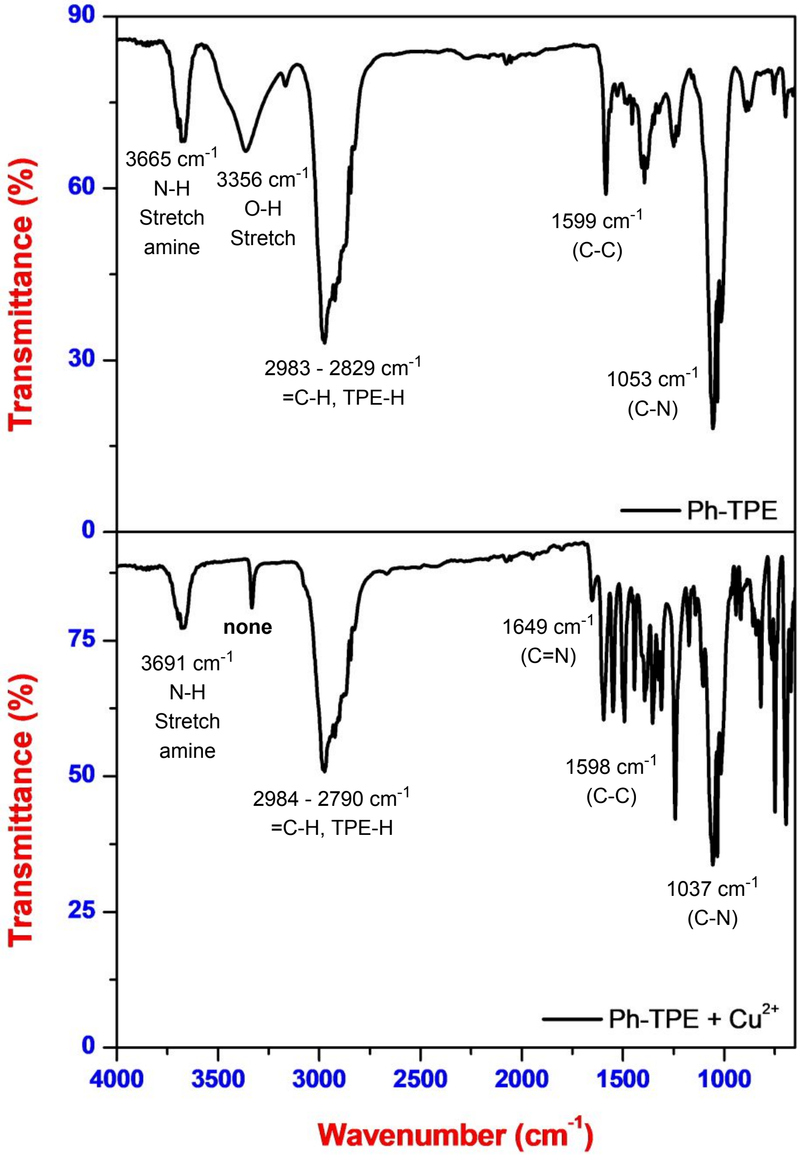

Figure S13. FTIR spectras of TPEs and TPEs-Cu^{2+}.

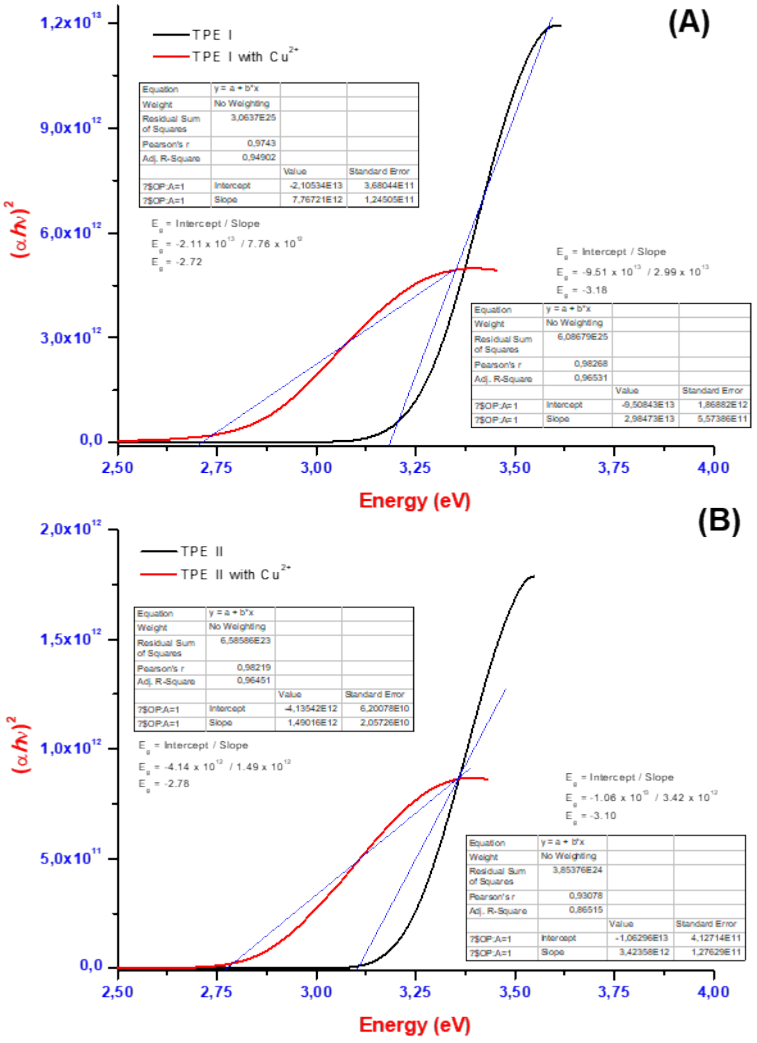

Figure S14. The band-gap energies details of (**A**) TPE **I** / TPE **I**-Cu^{2+} and (**B**) TPE **II** / TPE **II**-Cu^{2+}

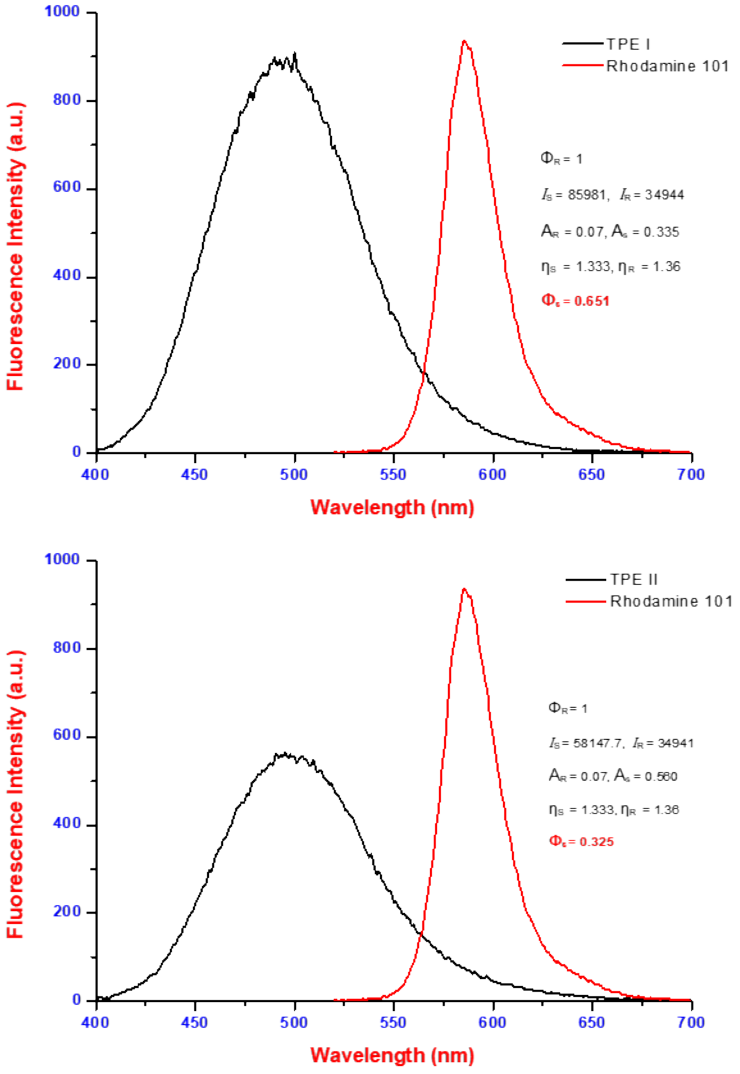

Figure S15. Fluorescence spectras of TPEs and rhodamine 101 for the fluorescence quantum yield

References

1. Toprak, M.; Lafzi, F.; Bayindir, S. Water-ratio directed selective turn-on fluorescence detection of copper and mercury in acetonitrile. *J. Photochem. Photobiol. A.* **2021**, 418, 113418.
